# Lithospheric double shear zone unveiled by microseismicity in a region of slow deformation

**DOI:** 10.1038/s41598-022-24903-1

**Published:** 2022-12-06

**Authors:** Rita de Nardis, Claudia Pandolfi, Marco Cattaneo, Giancarlo Monachesi, Daniele Cirillo, Federica Ferrarini, Simone Bello, Francesco Brozzetti, Giusy Lavecchia

**Affiliations:** 1 DiSPuTer, University G. d’Annunzio, Via dei Vestini 31, 66100 Chieti, Italy; 2 CRUST-Centro inteRUniversitario per l’analisi SismoTettonica tridimensionale, Chieti, Italy; 3 INGV Istituto Nazionale di Geofisica e Vulcanologia, c/o Centro Funzionale Protezione Civile-Regione Marche Centro Pastorale “Stella Maris” Via di Colle Ameno, 5, 60126 Torrette di Ancona, Italy

**Keywords:** Natural hazards, Solid Earth sciences

## Abstract

The deformation style of the continental lithosphere is a relevant issue for geodynamics and seismic hazard perspectives. Here we show the first evidence of two well-distinct low-angle and SW-dipping individual reverse shear zones of the Italian Outer Thrust System in Central Italy. One corresponds to the down-dip prosecution of the Adriatic Basal Thrust with its major splay and the other to a hidden independent structure, illuminated at a depth between 25 and 60 km, for an along-strike extent of ~ 150 km. Combining geological information with high-quality seismological data, we unveil this novel configuration and reconstruct a detailed 3D geometric and kinematic fault model of the compressional system, active at upper crust to upper mantle depths. In addition, we report evidence of coexisting deformation volumes undergoing well-distinguished stress fields at different lithospheric depths. These results provide fundamental constraints for a forthcoming discussion on the Apennine fold-and-thrust system's geodynamic context as a shallow subduction zone or an intra-continental lithosphere shear zone.

## Introduction

Outcropping or near-surface active thrust faults may propagate to depth with different structural styles, e.g., thin-skinned versus thick-skinned. They may or may not penetrate the basement, reach the lower crust, and even the upper mantle along localized shear zones^1–3^. These configurations are eventually supported by observations of the deep crust and shallow mantle reflectors dislocation and earthquake data. However, the accurate definition of the deep deformation style and its link with the shallow one is not always straightforward. It is particularly difficult in the case of low-seismicity levels, lack of proper monitoring systems, and/or inaccurate locations of seismic events. Hence, characterizing the geometry and kinematics of these regions is challenging, especially in areas with low deformation rates and blind onshore or offshore thrusting.

**Figure 1:**
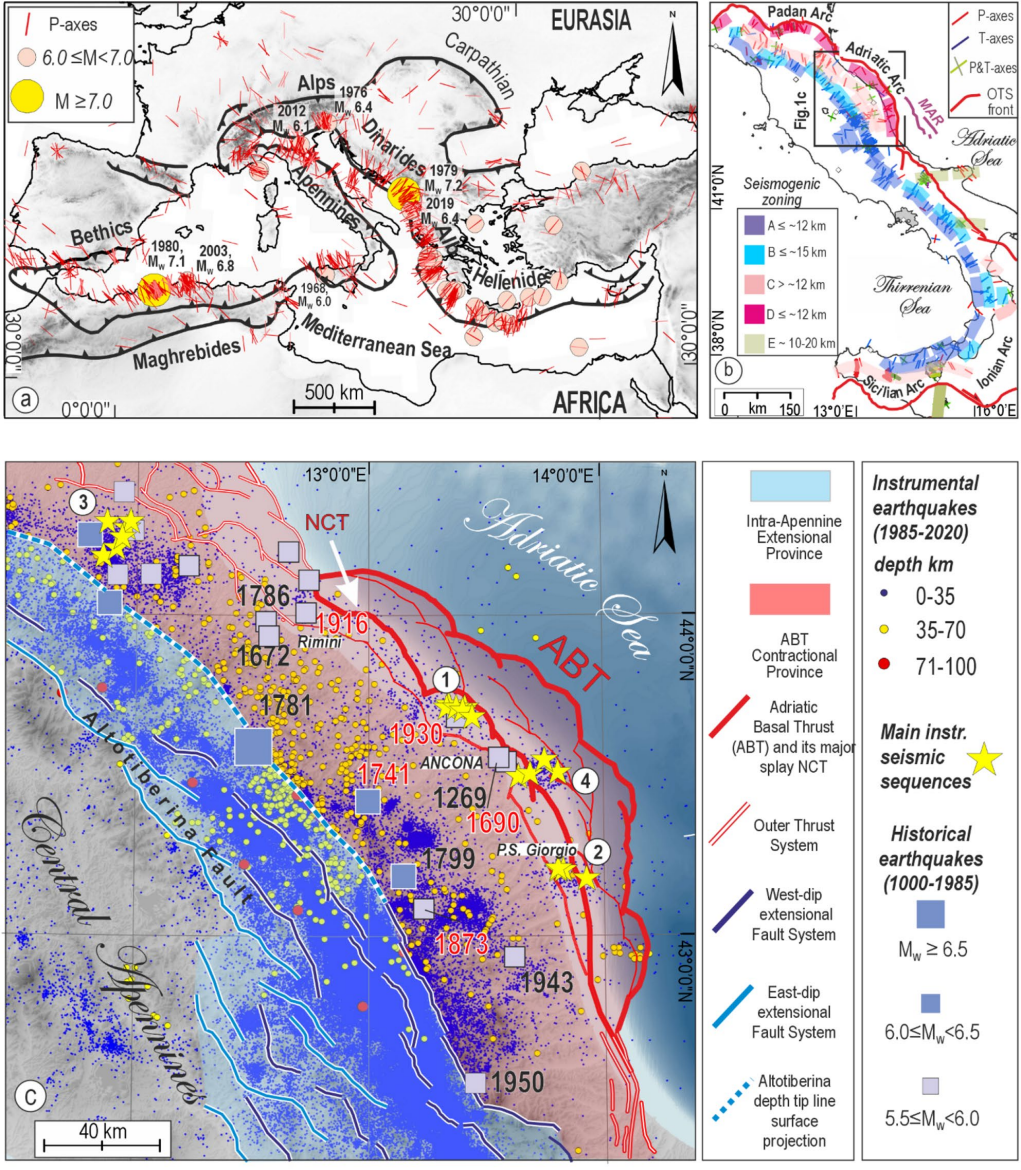
Seismotectonic sketch of eastern Central Italy in the Mediterranean framework. (**a**) Major thrust belts and distribution of P-axes of reverse/reverse-oblique earthquakes (M ≥ 3.0, depths < 40 km) occurred in the Mediterranean area in the time interval 1962–2016 (World Stress Map database^5^). (**b**) Kinematically homogeneous active deformation bands^15^ with P–T axes from a compilation of focal mechanisms (time interval 1968–2018, M_W_ ≥ 3.5, depths < 40 km): A = upper crust extension, B = mid-crust extension, C = lower-crust contraction; D = upper-crust contraction, E = mid-to-lower crust strike-slip. Key: MAR = Mid Adriatic Ridge. (**c**) Quaternary and potentially seismogenic extensional and contractional structures and tectonic domains in eastern Central Italy. Historical and instrumental earthquakes from Parametric Catalogue of Italian Earthquakes, CPTI15 v4.0^26,30^ and the Italian Seismological Instrumental and parametric Database ISIDe^28^ are also reported. The thick red lines represent the Adriatic Basal Thrust, and its major splay (Near Coast Thrust, NCT) analyzed in this paper; the thin red lines are the minor splays of ABT. The red labels represent the earthquakes cited in the text, and the yellow stars the major events that occurred in instrumental time; the numbers are related to the main seismic sequences of the study area. Key: 1 = Ancona 1972, M_W_ 4.8; 2 = Porto San Giorgio 1987, M_W_ 5.1; 3 = Faenza 2000, M_W_ 4.9; 4 = Ancona 2013, M_W_ 5.2.

In the circum-Mediterranean and Alpine–Hellenides fold-and-thrust belt, seismogenic compression prevailingly occurs at crustal depths (< 40 km; ISC-EHB Bulletin^4^) and with a radial pattern of P-axes perpendicular to the long-term structural trends^5^ (Fig. 1a). At depths from 40 to 70 km, subordinate sub-crustal seismicity is observed all over the belt^6,7^. Conversely, there is no intermediate seismicity (70–300 km), apart from the Benioff plane offshore Calabria and Hellenides Arc, where the seismicity reaches ~ 600 and ~ 300 km, respectively^8–10^.

In the last 40 years, thrust earthquakes with M_W_ > 6.0 were released at (1) upper crustal depths (< ~ 10–12 km) in the Maghrebide domain (El Asnam 1980, M_W_ 7.1 and Zemmouri 2003, M_W_ 6.8), in the Northern Apennines of Italy (Emilia 2012, M_W_ 6.1), and the eastern Alps (Friuli 1976, M_W_ 6.4) and (2) at lower crust depths (20–30 km), along the Dinarides-Albanides system (Montenegro 1979, M_W_ 7.1, Durres 2019, M_W_ 6.4) and in western Sicily (Belice earthquake 1968, cumulated M_W_ 6.1) (Fig. 1a). In Italy, seismogenic compression at lower crust depths is also highlighted by background seismicity associated with the Adriatic Basal Thrust (ABT) and the Sicilian Basal Thrust (SBT)^11,12^, as well as in Northern Italy^13,14^ (Fig. 1b).

In the cases of the coexisting and syn-kinematic upper crust and lower crust seismicity within the boundary of the same seismotectonic domain, a question concerns whether the shallow and the deep seismogenic deformations are physically connected along distinct crust-scale shear zones. with evident implications for seismogenic potential.

Recent advances in earthquake monitoring often allow the reconstruction of complex fault systems, unveiling new or incipient seismogenic sources, and eventually recording background seismicity that reliably illuminates geological structures.

Here, we present a new high-resolution seismological dataset of relocated earthquakes and focal mechanisms in eastern Central Italy, revealing two seismogenic and well-distinguished lithospheric-scale shear zones. The first refers to the ABT^11^, a crustal-upper mantle thrust zone, and the second one to a hidden SW-dipping lithospheric thrust located beneath the ABT at depths between 20 and 60 km. Further, we give a faithful reconstruction of the geometry and kinematics of these thrusts and define the stress field acting on the area providing important elements for the discussion on the debated geodynamic context. To this aim, we adopt a multi-scale approach and (1) analyze the geometry and kinematics of earthquakes belonging to two well-distinct and closely spaced lithospheric-scale seismic volumes, (2) calculate the corresponding 3D-stress tensor, and (3) build 3D non-planar geometric-kinematic fault models integrating earthquake and geological data.

## Seismotectonic framework

The ABT is a segment of the Outer Thrust System of Italy (OTS) (Fig. 1b,c) representing the outermost and still active front of the Apennine fold-and-thrust belt; the latter progressively developed since late Miocene times together with the opening of the Tyrrhenian system in its rear^15^. The OTS is hierarchically articulated in two major arcs (Fig. 1b), the Padan-Adriatic and Sicilian-Ionian ones. In turn, the Padan-Adriatic arc is organized in three outwards convex arcs^16^, the ABT being the southernmost one. In the central Adriatic, a more external late Pliocene–Quaternary contractional deformation is represented by the mid-Adriatic Ridge (MAR)^17^ (Fig. 1b).

The upper crust geometry of ABT and its hanging wall splays consist of WNW–ESE- to NNW–SSE-trending en-échelon folds and corresponding thrusts deforming the Meso-Cenozoic multilayer and occasionally penetrating across the basement (Fig. 1c). In addition, subordinate N–S right-lateral and E–W left-lateral strike-slip faults are present. The CROP-03 near-vertical reflection profile reveals the ABT down-dip prosecution to the bottom of the Moho^18,19^. Several regional thrusts are present at the ABT hanging wall, the most continuous and relevant one running offshore not far from the coastline (Near Coast Thrust, NCT, Fig. 1c).

The contractional deformation associated with OTS has been active since late Pliocene times and is contemporaneous with the nearly coaxial extensional one observable along the axis of the Apennine Chain^20^ (Fig. 1b). The latter is mainly achieved by high-angle westward-dipping normal faults, detaching on eastward- dipping low-angle basal planes (for example, the Altotiberina fault, ATF, e.g., Refs.^21–24^), well-known and supported by geological and geophysical data.

Different from worldwide seismicity, characterized by more energetic events in thrust zones^24^, the Italian Contractional seismotectonic Province^15^ shows widespread seismicity and moderate earthquakes, rarely exceeding M_W_ 6.0–6.5, with a deformation rate between 1 and 3 mm/year^25^. Conversely, the Apennine Extensional Province^15^ is characterized by high seismicity rates with events up to M_W_ 7.0–7.5^26,27^ and a GPS velocity up to 5 mm/year^25^.

The Apennine normal fault earthquakes are mainly located at upper crustal depths (< 12–14 km), whereas the reverse and reverse-oblique fault ones deepen from upper crust depths (< ~ 10–12 km) along the coastal Adriatic area to lower crust depths (~ 20–30 km) in the Apennine Foothills region and upper mantle depths (~ 60–70 km), beneath the Apennine Extensional Province^28^ (Fig. 1b and Supplementary Figs. S1–S3a). The evidence of two geographically well-distinct depth ranges for the thrust-related seismicity has led to the identification of two broad seismotectonic provinces, each of relatively homogenous deformation, referred to as Shallow and Deep Contractional provinces identified in Central Italy and Sicily^11,15,18,29^. Within the study area, the Shallow Province extends from the ABT near-surface trace to its 10 km-depth contour line; the Deep Province extends from 10 km to about 25 km within the Apennine Foothills region and extends further west to upper mantle depths (~ 60 km), beneath the Extensional Apennine Province.

In instrumental times, the Contractional Province is characterized by a few seismic sequences, never exceeding M ~ 5.0. They occur at upper crustal depths along the coastal area (e.g., Ancona 1972, M_W_ 4.8; Porto San Giorgio 1987, M_W_ 5.1; Ancona 2013, M_W_ 5.1) and by minor swarm-like seismic sequences, occurring about 50 km west to Porto San Giorgio, at a depth of ~ 20–30 km beneath the Apennine Foothills region (Fig. 1c and Supplementary Fig. S3). In historical times, earthquakes with M_W_ up to ~ 6.0^30^, possibly associated with ABT and its northward continuation, were released both along the coastal area (e.g., Conero offshore 1690, M_W_ 5.9; Rimini 1916, M_W_ 5.7; Senigallia 1930, M_W_ 5.9) and the Foothills area (e.g., Fabriano 1741, M_W_ 6.2; Sarnano 1873, M_W_ 6.0)^31^ (Fig. 1c and Supplementary Fig. S3b).

## Datasets

### EQS-catalog

**Figure 2:**
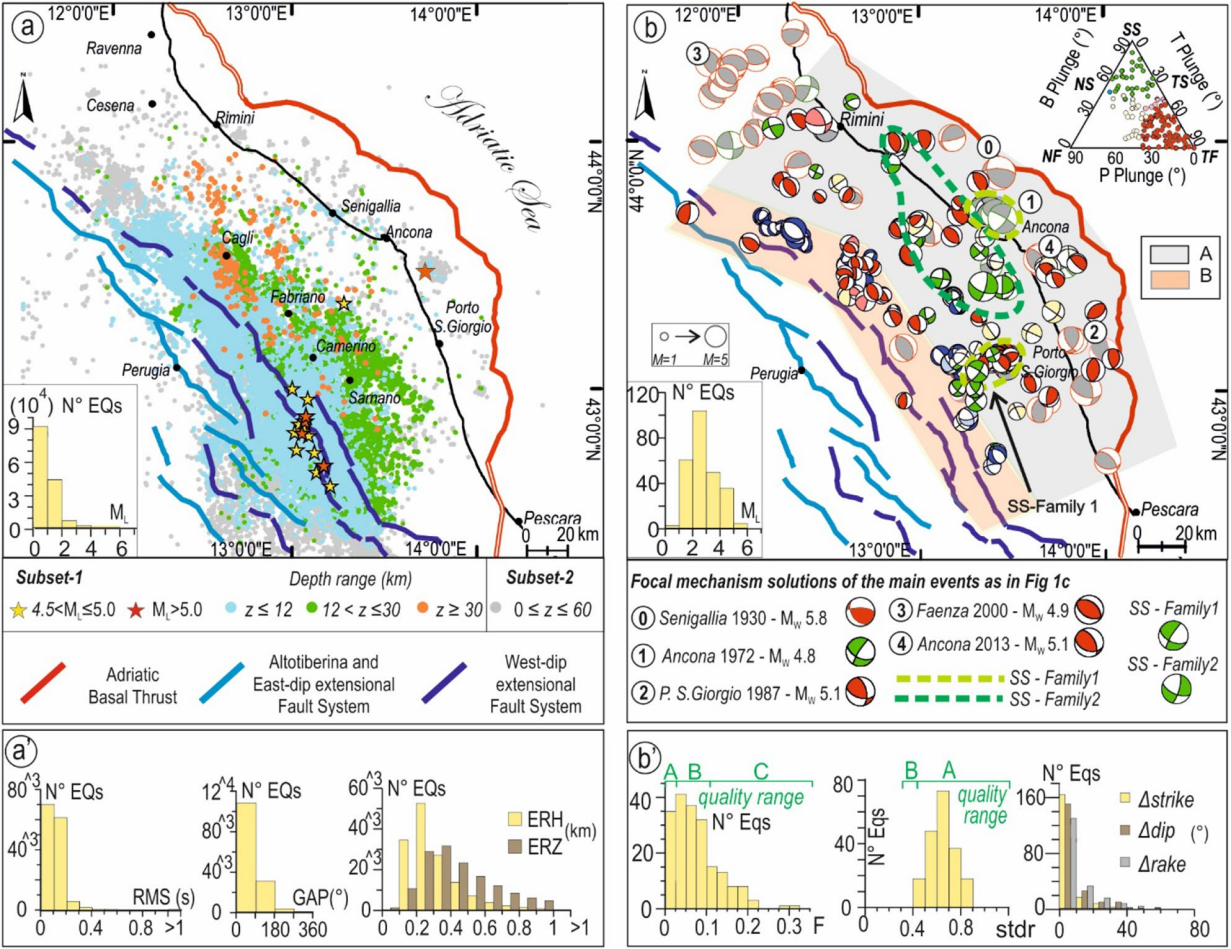
Map view of the earthquakes and focal mechanisms computed and used for this study (EQS- and FMS-Catalogs) (Generic Mapping Tools, GMT 6.4.0, https://www.generic-mapping-tools.org/). (**a**) Relocated earthquakes occurred in eastern Central Italy from 2009 to 2017 (0.0 ≤ M_L_ ≤ 5.8) calculated using the recordings of the ReSIICO seismic network and a 3D velocity model^33^. The colored and grey dots represent the higher-quality (Subset-1, EQS-Catalog_HQ.txt in supplementary material) and the lower-quality (Subset-2, EQS-Catalog_LQ.txt in supplementary material) subsets, respectively, as described in the text and supplementary material; the bottom-left inset represents the histogram of the magnitude distribution. (**a′**) Histograms of the summary of quality parameters of Subset-1. Key: RMS = root mean square of residuals of the final location, GAP = maximum azimuthal gap, ERH = maximum horizontal error, ERZ = maximum vertical error. (**b**) New (colored beachballs with black border) and collected (grey beachballs with red border) fault plane solutions scaled with magnitude and their respective kinematics classification^41^ (triangular diagram in the top right corner). The beachballs color code is red for reverse/reverse-oblique faults, blue for normal/normal oblique faults, green for strike-slip faults and light yellow for unknown kinematics. A and B represent the polygonal areas used to select FMs within different depth ranges (A from 0 to 70 km, B from 12 to 70 km, see detail in “FMS-Catalog” and Supplementary Fig. S9). The dashed light and dark green lines indicate the areas where we observe strike-slip solutions with different P-axis orientations. The bottom-left inset represents the histogram of the magnitude distribution of the new focal mechanisms. (**b′**) Histograms of the quality parameters F, stdr, ∆strike, ∆dip, and ∆rake as given in FPFIT code^40^.

We relocated ~ 170,000 seismic events^32,33^ (0.0 ≤ M_L_ ≤ 5.8) that occurred in eastern Central Italy from 2009 to 2017 between the ATF and the ABT (Fig. 2a), expression of both extensional and contractional tectonics.

The events were recorded by the regional network ReSIICO^34^ integrated with the Italian seismic network (RSN), which comprises 103 seismic stations allowing good coverage of eastern Central Italy, with velocimeters and accelerometers deployed along the coastal area (Supplementary Fig. S4). These locations (EQS-Catalog in supplementary material) were derived from developing a mixed automatic-manual seismic catalog for eastern Central Italy^35,36^. We used P- and S-phases detected by human operators (period 2009–2013) and by the automatic RSNI-Picker (period 2014–2016) and its updated version (2017)^37^. The events for which the automatic procedure could not produce a good-quality location were manually revised (Supplementary Text S1).

The EQS-Catalog was divided into two subsets based on the quality of the final location, accounting for a summary of statistical parameters, specifically on the distance between the maximum likelihood and expectation hypocentre locations representing a good estimate of the stability of the inversion.

Subset-1 contains 83% of EQS-Catalog data (~ 141,051 events) and consists of high-quality seismic locations (horizontal and vertical formal error never exceeding 1 km) (Fig. 2a'). Subset-2 contains the remaining 17% of data (~ 27,941), which, although of lower quality, are still relatively stable, as shown in the statistical distribution of the location parameters given in Supplementary Figs. S5 and S6.

We show, in Fig. 2a, the epicentral distribution of EQS-Catalog highlighting the characteristics of Subset-1 and displaying Subset-2 as background. In Supplementary Figs. S7 and S8, the two subsets are represented in section view.

The EQS-Catalog has a completeness magnitude of ~ M_L_ 0.9. The maximum concentration of events (~ 96%) occurs within the boundaries of the Apennine Extensional Province, at depths < 12–14 km (Fig. 1c). The 2016–2017 Central Italy Seismic Sequence (Amatrice-Visso-Norcia^38,39^, M_W_max 6.5) largely enhanced the number of events in the area. The remaining 4% is located within the boundaries of the Shallow and Deep Contractional Province; it has a completeness magnitude of ~ M_L_ 1.10 ± 0.09 and consists of events with 0.0 ≤ M_L_ ≤ 4.8 deepening westward from upper-crust depths along the coastal Adriatic area, to lower crust depths in the Foothills region and upper mantle depths (~ 60 km) beneath the Apennines (Fig. 2a).

### FMS-Catalog

The re-picking process allowed us to collect a large number of P-wave polarities and compute 115 new focal mechanism solutions with FPFIT algorithm^40^ (FMS-Catalog in supplementary material) associated with the Contractional Province. It consists of events with 1.4 ≤ M_L_ ≤ 4.8 having more than 20 precise observations homogenously distributed on the focal sphere (Fig. 2b). The quality factors (Q) are described in Fig. 2b'. In particular, the FMS-Catalog contains the solutions of the events located (1) at depths between 0 and 70 km within a polygon enclosed between the trace of the ABT front and the outer front of the Extensional Province, the latter corresponding to the surface projection of the ATF deep tip line (area A in Fig. 2b and Supplementary Fig. S9), (2) between 12 and 70 km within a neighboring polygon corresponding to the outer sector of the Extensional Province (area B in Fig. 2b and Supplementary Fig. S9).

The FMS-Catalog is given as supplementary. Each event also reports the kinematic classification according to six classes (NF, NS, SS, TF, TS, and UK^41^) (see the triangular diagram in Fig. 2b), and the association with the major fault structures, as later identified.

Whenever possible, to further check the quality of our focal solutions, we compared them with the ones obtained for the same events with other methods (i.e., TDMT^42^, RCMT^43^) or with a 1D velocity model^34^ (Supplementary Fig. S10). The comparison reveals very similar solutions and reinforces the robustness of the focal mechanisms of our catalog.

In Fig. 2b, the FMS-Catalog is further integrated with other 65 focal mechanisms that occurred in the previous time interval (1967–2009) and derived from the literature^42,43^.

The overall focal mechanisms (180 events) mainly consist of prevailing reverse and reverse/oblique solutions, with nearly horizontal P-axes rotating from SW–NE to WSW–ENE to W–E (76%) and subordinate strike-slip (SS) FMs belonging to two families (Fig. 2b). One (SS-Family1) consists of events characterized by SW–NE trending P-axes, coaxial to the ones of reverse/reverse-oblique solutions (16%). They are mainly located near the town of Ancona at upper crust depth (i.e., Ancona 1972 earthquakes) and in the Apennine Foothills region at lower crust depth. The other family (SS-Family2) consists of lower crust events with NNW-SSE trending P-axes (6%) located from Ancona to Rimini along the coastline.

## Results

### 2D analysis of earthquake/fault association

**Figure 3:**
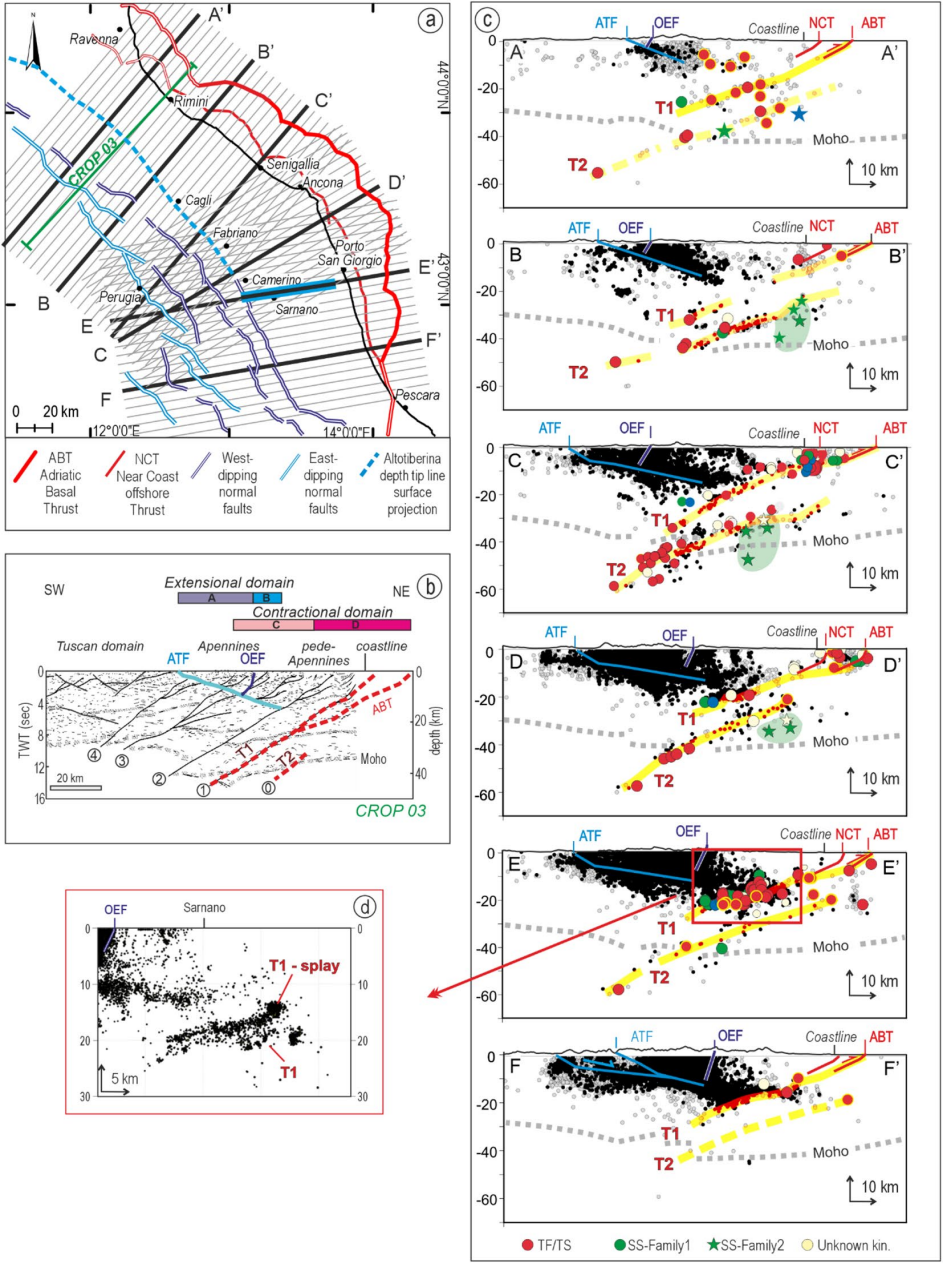
Hypocentral cross-sections and inferred earthquake-fault association (Data from EQS- and MFS-Catalogs in this paper). (**a**) Map-view traces of 70 narrowly spaced radial cross-sections and six regional transects (A–F) used to build the 3D fault model. (**b**) Line-drawing of the near-vertical CROP-03 seismic reflection line across the northern sector of the ABT^18^; thrust numbered from 4 to 1 progressively rejuvenate in an age moving eastward from middle Miocene (thrust 4) to late Pliocene–Quaternary (thrust 1); T1 and T2 as in (**c**); (**c**) Section-view projection (within 20 km of the trace line) of earthquakes and focal mechanisms from this study (EQS- and FMS-Catalogs ), plus focal solutions in the 1967–2009 time interval from the literature^42,43^ (e.g., yellow bordered circle). Key: black dots = Subset-1 data; grey dots = Subset-2 data; colored dots = focal mechanisms with kinematic color code as in Fig. 2b (Red = reverse, blue = normal, green = strike-slip, light yellow = unknown kinematics); green dots = strike-slip Family1 with SW–NE P-axes trending; green stars = strike-slip Family2 (deep-SS) with NNW–SSE P-axes trending; OEF = trace of Outcropping west-dipping Extensional Front. The yellow lines enveloping hypocenters and focal mechanisms offer a section view of the ABT (T1) and the underlying hidden lithospheric thrust identified in this paper (T2); the Moho depth line is from Di Stefano et al.^69^. (**d**) Detail from transects E showing the intersection zone between T1 and T1-splay; the hypocenters from EQS-Catalog are projected with a half-width of 2.5 km.

The depth distribution of the data from the EQS- and FMS-Catalog (2009–2017), integrated with focal mechanisms from the literature (1967–2009), was analyzed in 2D view and projected along 70 radial cross-sections, organized in three sets (N040°, N060°, and N080° directions), along 23 parallel N055° striking cross-sections and 6 regional transects (Fig. 3a,c, and Supplementary Figs. S11–12). In addition, geological and geophysical cross-sections available in the literature (Supplementary Fig. S11) were used to reconstruct the near-surface traces of the major fault alignments necessary to correlate the geological structures with the seismicity distribution.

The section-view earthquake distribution and kinematics show a complex pattern (Fig. 3a,c, and Supplementary Fig. S12). The western side of each regional transect is characterized by a large concentration of events (about 96% of data from the EQS-Catalog). They are distributed in an east-deepening wedge-shaped seismogenic volume and represent the upper crust extensional domain not investigated in this paper but well known in the literature^22,23,44,45^. They are associated with the east-dipping ATF and the antithetic west-dipping high-angle normal faults.

The number of earthquakes on the eastern side of the transects substantially decreases. Notwithstanding, the events depict two well-distinct west-deepening seismogenic volumes, hereinafter called T1 and T2, with predominant reverse and reverse/oblique kinematics and subordinate strike-slip solution (SS-Family1). Notably, such strike-slip focal mechanisms, with P-axes coaxial with the ones of reverse/reverse-oblique types, mainly concentrate at lower crust depth along transect E (Fig. 3c).

T1 develops at a low angle (~ 20°) along the down-dip prosecution of the ABT front to a depth of ~ 35 km (Fig. 3b,c). A major splay corresponds to the Near Coast Thrust (NCT, Figs. 1c and 3). T1 and T1-splay intersect at depths of ~ 20 km, as evident in the hypocentral detail of transect E given in Fig. 3d. T2 is systematically located beneath T1, with a similar average dip-angle (~ 20°), at depths between ~ 20 and 60 km (Fig. 3c).

The regional transects also show an independent deformation volume (SS-Family2, called deep-SS), with prevalent strike-slip deformation, located beneath T2 at a depth greater than ~ 25 km (green stars in Fig. 3c).

**Figure 4:**
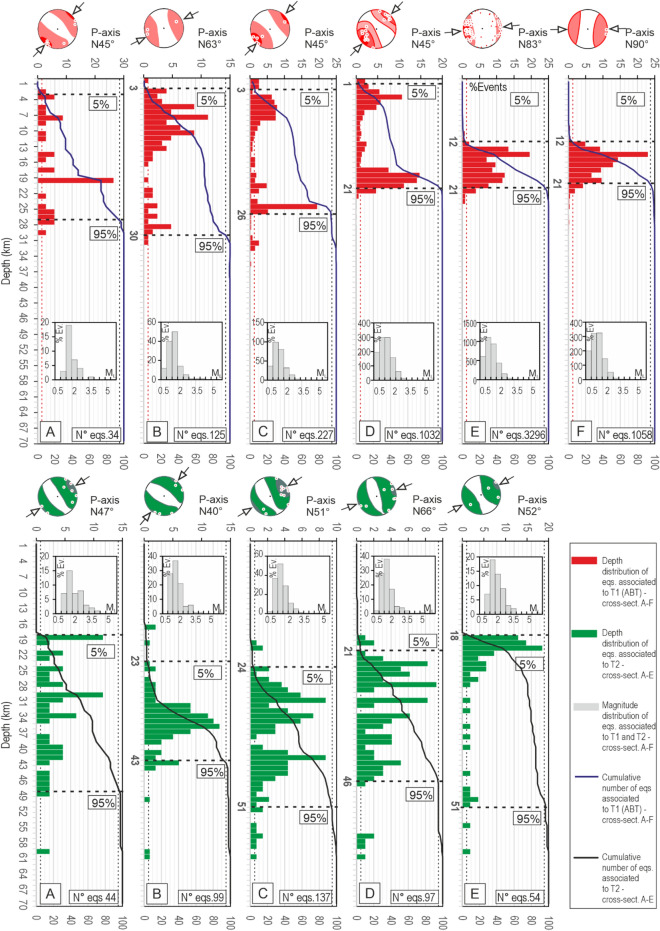
Depth distribution of events associated with T1 (red histograms) and T2 (green histograms) along the six transects (A–F) in Fig. 3c; in each histogram, the black horizontal dashed lines identify the top and bottom of the seismogenic layer assumed to correspond to 5% and 95% of the hypocentral distribution, respectively. The small grey histograms represent the magnitude distribution of events within each transect. The stereoplots represent the density contour of the P-axes (white circles) of the focal mechanisms falling within each transect.

Figure 4 summarizes the depth distribution and kinematics of the events of the EQS- and FMS-Catalogs associated with T1 and T2, as projected along transects A–F. The hypocentral distributions associated with T1 present a well-evident bimodal pattern along transects B, C, and D, with maxima concentration of events at upper crust depths (< 10 ± 2 km) and distributed seismicity at lower depths down to ~ 30 km. Only relatively deep events characterize the eastern sector of transects E and F, where almost all the compressional seismicity in the analyzed time interval, that prevailing consists of low magnitude events (mode of magnitude distribution ~ 0.8–0.9 in Supplementary Fig. S13), is concentrated at depths between 12 and 21 km. The stereoplots above the histograms represent the density contour of P-axes orientation derived from the FMS-Catalog; it clearly shows a clockwise rotation of the average P-axis from transects A to F.

The events associated with T2 are concentrated at a prevailing depth between 20 and 50 km, with few events up to 60 km (less than 5%). Differently from P-axes associated with T1, the orientation of P axes representing the deformation along T2 is relatively stable, showing a slight rotation of ~ 30° from north to south (transect B–E).

Based on the earthquake distribution, T1 coincides with the location of ABT^11^. T2, differently, was unknown in this configuration, and we advance it may be an independent thrust having regional extent.

To validate the association of T1 with ABT, and T2 with unveiled thrusting beneath it and to constrain their geometries and structural style with independent geological and geophysical data (Fig. 3b), we report a line drawing of the CROP-03 near-vertical section^18,46^, which runs close to transects A and B and is helpful to perform the structural interpretation of the earthquake depth distribution. The CROP-03 has an SW-NE direction across the Tyrrhenian thinned crust, the Apennine and Apennine Foothills thickened crust, and the Adriatic foreland. It shows four major SW-dipping regional thrusts (i.e., 1–4 in Fig. 3b) penetrating the entire crust and dislocating the Moho. The thrusts and their hanging wall fold-and-thrust structures progressively rejuvenate in age from the middle Miocene (thrust 4), Late Miocene (thrust 3), early Pliocene (thrust 2), and late Pliocene–Quaternary (thrust 1, i.e., T1-ABT). In a more external position, at Moho depths within the Adriatic foreland (thrust 0; Fig. 3b), is evident a thrust discontinuity which looks to correspond to T2, as in Finetti et al.^47^.

### Strain and stress analysis

**Figure 5:**
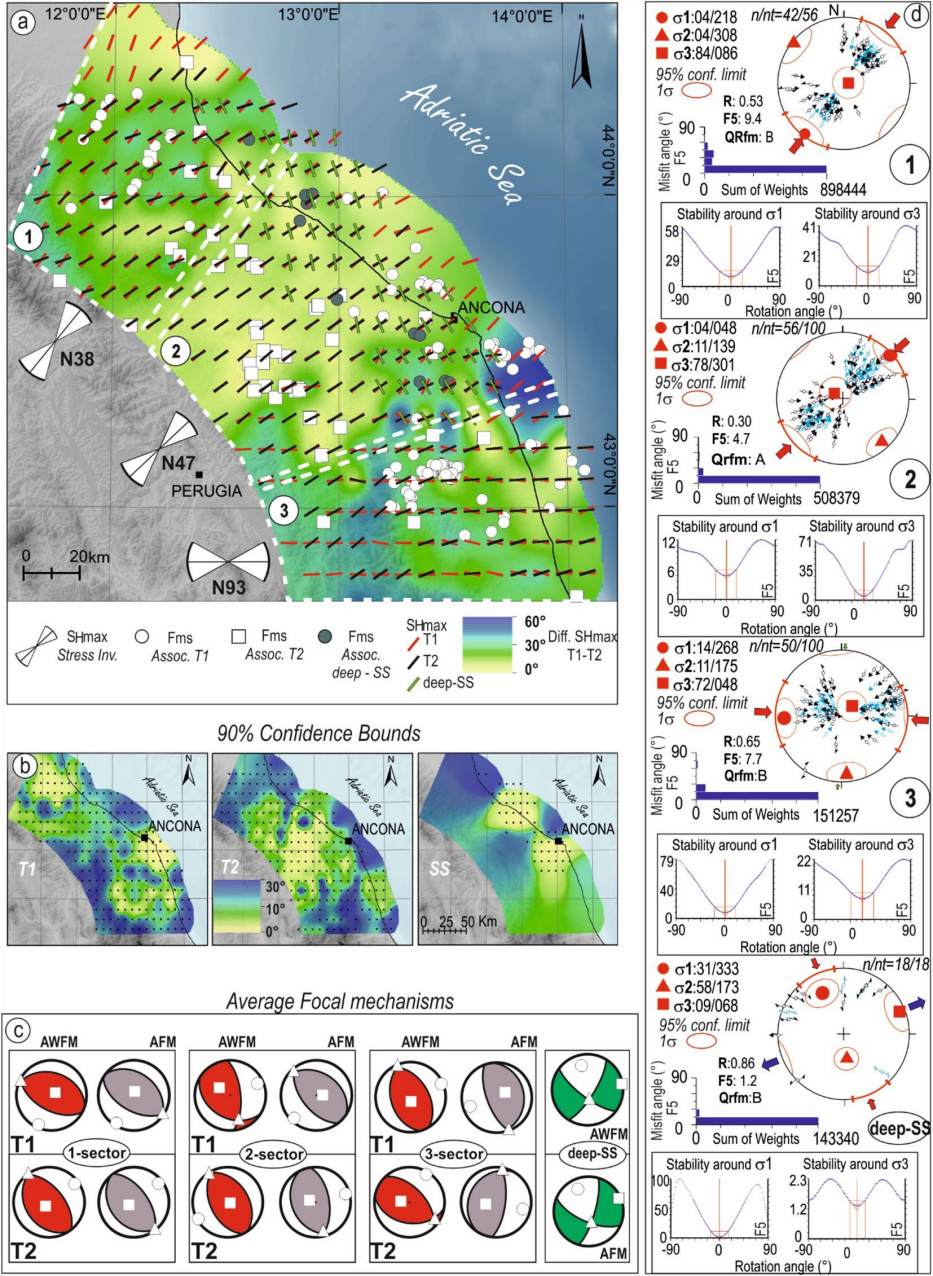
Strain and stress fields of the study area retrieved from the focal mechanisms shown in Fig. 2b and Table 2. (**a**) Map view of SH_max_ distributions computed from the FMs associated with T1 (red bar), T2 (black bar), and SS (green bar) based on the event location (Fig. 3c). The interpolation of SH_max_ for T1, T2, and SS was performed following the approach of Carafa and Barba^38^. The circle and square symbols represent the FMs used for the analyses, and the white dashed lines represent the map-view boundary of the sectors (1–3) into which the study area has been divided. The colored map underlying SH_max_ represents the differences, in degree, between T1 and T2 axes (from yellow shades-to-blue tones: good-worst); the left-side white bowties indicate the direction of the maximum horizontal compressive stress, (azimuthal SH_max_) for the three sectors as derived from the stress inversion. (**b**) Maps representing the interpolation quality for T1, T2, and deep-SS datasets. (**c**) Average focal mechanisms of T1 and T2 within sectors 1–3 and of deep-SS, beneath T2; the average solutions were computed using the Bingham statistics (AFM, red beachballs) and the moment tensor summation (data weighted with the magnitudes, AWFM, purple beachballs). Key: circle = P-axis; square = T-axis; triangle = B-axis. (**d**) Stress inversion results from focal mechanisms for compressional sectors 1, 2, and 3 and strike-slip of SS-Family2, carried out following the inversion procedure as in Delvaux and Sperner^49^. The black and light blue arrows indicate the observed and theoretical slip directions, respectively. Histograms represent the corresponding distribution of the misfit function versus the number of observations; nt = total number of data; n = number of successfully inverted data; σ1, σ2, σ3 the principal stress axes; R the stress ratio (σ2–σ3)/(σ1–σ3); the quality ranking factors (Qrfm) is based on the World Stress Map ranking criteria^5^; the diagrams below each stereonet show the stability of the objective function (**F5**)^47^ around σ1 and σ3. The stress inversion parameters with associated uncertainties are listed in Table 2.

The new FMs and the ones collected from the literature were analyzed to reconstruct the strain and stress fields acting on the crustal volumes of the study and surrounding areas (Fig. 5a–c) and define the kinematics of T1, T2, and the T2 footwall volume characterized by strike-slip solutions (deep-SS).

We preliminarily computed^48^, on a regular grid along T1, T2, and deep-SS, the maximum horizontal stress orientation (SH_max_)^41^. The distribution of SH_max_ along with T1 (red bars in Fig. 5a) shows an average direction rotating clockwise (N045° to N090°) from north to south. A less evident rotation of SH_max_ (N050° to N080°) is instead observed along T2 (black bars in Fig. 5a), due to the lack of information in the southern sector of the study area (only four FMs are available). Moreover, a localized and homogeneously distributed SH_max_ (N160°) characterizes the strike-slip deformation at the footwall of T2. The reliability of the interpolation of SH_max_ for the T1, T2, and deep-SS data sets is shown in Fig. 5b and mainly reflects the number of data associated with the three domains.

T1 and T2 show a coaxial trend of SH_max_, above all in the central sector. It is evident from the background map of Fig. 5a,b, where the colors represent the differences in the SH_max_ orientation between the two planes at each grid point and the interpolation quality for T1, T2, and deep-SS datasets, respectively.

Based on SH_max_ orientation, we identified three sectors (1 = northern sector, 2 = central sector, and 3 = southern sector; Fig. 5a) in which SH_max_ can be considered homogeneously oriented.

Within these three sectors, we computed the average focal mechanisms (Fig. 5c) using the Bingham statistics (AFM) and the moment tensor summation by weighting the data with the magnitudes (AWFM). The resulting FMs show the dominant kinematics of each domain: reverse/reverse-oblique for T1 and T2, and strike-slip for deep-SS (Table 1). Significant differences between AFM and weighted AWFM are shown only for sector 3 and offshore, south of Ancona.

**Table 1:** P, T, and B axes of average focal mechanisms and weighted average focal mechanisms (W) associated with T1, T2, and deep-SS related to sector1 = 1, sector2 = 2, sector3 = 3; ^a^Average focal mechanisms computed without considering the Ancona 1972 focal mechanisms.

		Average focal mechanisms											
		P-trend (°)	P-plunge (°)	T-trend (°)	T-plunge (°)	B-trend (°)	B-plunge (°)	N° Tot	Depth-range (km)	Mmin	Mmax	N° (M < 4)	N° (M ≥ 4)
T1	1	214	9	11	80	123	4	16	8–27	3.5	4.8	5	11
	2	236	6	30	83	145	3	30	0–11	1.7	5.1	18	12
	2^a^	227	12	85	76	319	9	24	0–11	1.7	5.1	18	6
	3	267	16	34	66	171	18	70	2–22	1.5	5.1	61	9
T2	1	52	13	243	77	143	2	13	22–58	1.7	5.2	6	7
	2	74	12	289	75	166	8	29	21–56	1.4	4.4	28	1
	3	104	23	261	65	11	9	5	19–58	2.1	4.1	4	1
Deep-SS	SS	349	24	80	1	172	66	12	22–47	2.1	4.8	9	3
T1(W)	1	214	9	11	81	123	4	16	8–27	3.5	4.8	5	11
	2	64	17	305	59	163	26	30	0–11	1.7	5.1	18	12
	2^a^	52	7	144	19	302	69	24	0–11	1.7	5.1	18	6
	3	269	8	42	78	178	9	70	2–22	1.5	5.1	61	9
T2(W)	1	54	12	223	78	323	2	13	22–58	1.7	5.2	6	7
	2	241	4	106	85	331	4	29	21–56	1.4	4.4	28	1
	3	31	8	277	70	124	19	5	19–58	2.1	4.1	4	1
Deep-SS(W)	SS	332	28	66	6	168	61	12	22–47	2.1	4.8	9	3

The focal mechanisms belonging to sectors 1- to 3 and the deep-SS were independently inverted^49^ to define the stress-tensor acting in each sector. The inversion of focal mechanisms in sectors 1, 2, and 3 indicates a reverse faulting stress regime with nearly horizontal, NNE–SSW, NE–SW, and E–W trending σ1-axes (04/218, 04/048, 14/268) and sub-vertical σ3-axes (84/086, 78/301, 72/048), respectively; the shape factor is equal to 0.53, 0.30 and 0.65, respectively. The stress orientation solutions fall in the quality rank (QRfm) B, A, and B, respectively, as shown in Fig. 5d and Table 2. The stress tensor computed inverting the deep-SS focal mechanisms shows an NNW–SSE trending σ1-axis and a nearly-horizontal NNE–SSW σ3-axis. The solution has a (QRfm) B quality rank.

**Table 2:** Stress inversion parameters σ1, σ2, σ3, and R′ are computed for sectors 1, 2, and 3 starting from the FMS-Catalog and the collected focal mechanism solutions. Key: Sec. = sector; n/nt = number of inverted FMs with respect to the total number of data; σx Tr. = trend of σ1, σ2, σ3 axes; σx Pl. = plunge of σ1, σ2, σ3 axes; 1 σ = 1 σ standard deviation; QRfm = quality factor as in the World Stress Map^5^. R′ = R for normal faulting regime; R′ = 2 -R for strike-slip faulting regime; R′ = 2 + R for reverse faulting regime.

Sec	n/nt	σ1 Tr	σ1 Pl	± 1σ1	σ2 Tr	σ2 Pl	± 1σ2	σ3 Tr	σ3 Pl	± 1σ3	R′	± 1σ	QRfm
1	42/56	218	4	19	308	4	20	86	84	19	2.5	0.3	B
2	56/100	48	4	19	139	11	17	301	78	20	2.3	0.3	A
3	50/100	268	14	19	175	11	19	48	72	18	2.7	0.3	B
Deep-SS	18/18	333	31	19	173	58	15	68	9	20	1.1	0.5	B

### 3D fault model

**Figure 6:**
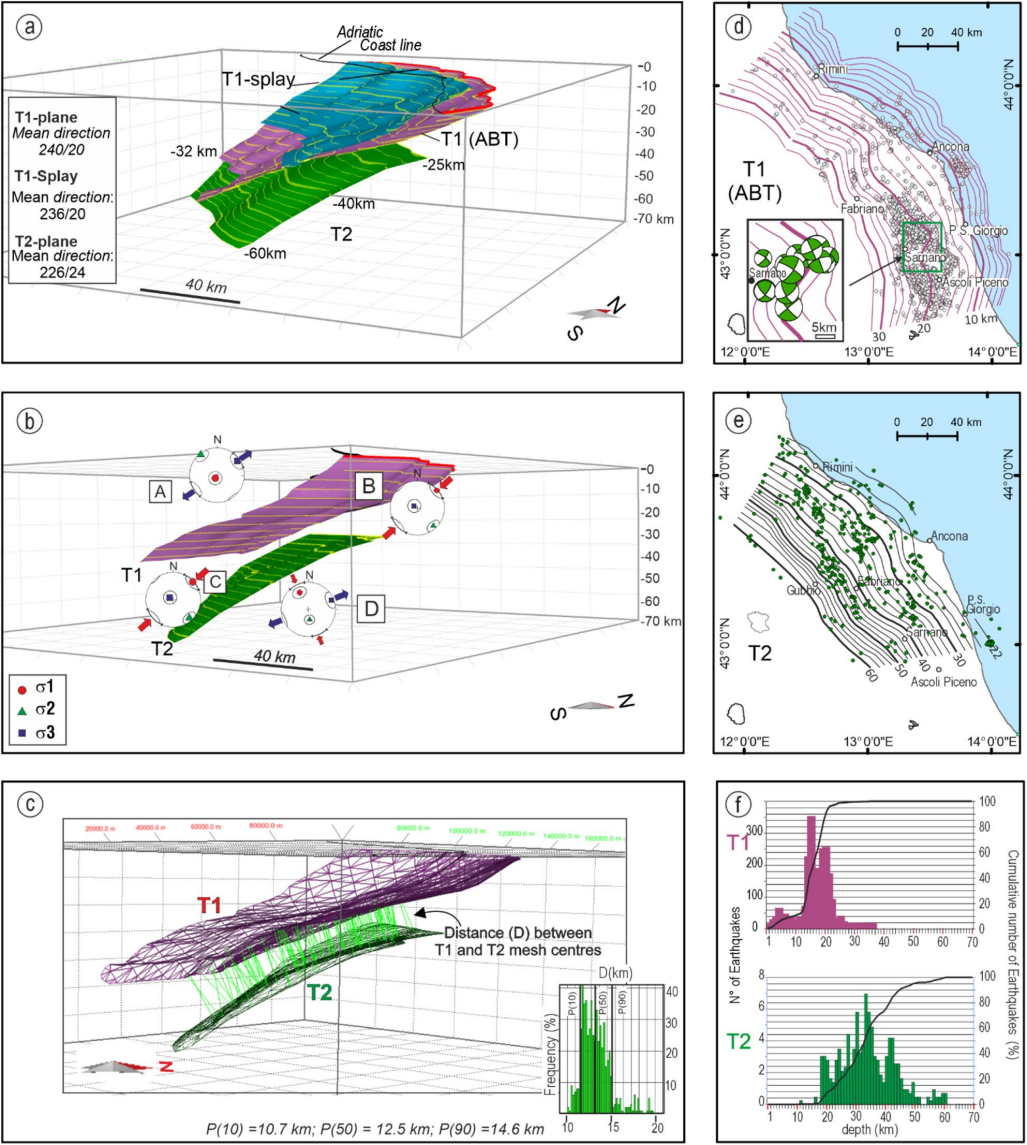
3D Fault model of the Adriatic Basal Thrust (T1), its regional splay (T1-splay), and the underlying hidden thrust (T2). (**a**) Fault surfaces built with the MOVE Suite software v. 2020.1 (Petroleum Experts Ltd), view from SE. (**b**) Fault model view from south and stereonet representation of the coexisting stress regimes at different crustal depths (A = upper to mid-crust tension; B = upper crust compression; C = lower crust compression, D = lower crust strike-slip). (**c**) distance between T1 and T2 measured with the Similar Construction Method of the MOVE Suite software v. 2020.1 (Petroleum Experts Ltd) tool; the histogram in the lower right corner represents the distance distribution between T1 and T2. (**d**,**e**) depth contour lines of T1 and T2, spaced 2.5 km along the depth, with the epicentral distribution of the events extracted from the EQS-catalog and associated with T1 and T1-splay (white dots) and T2 (green dots); (**f**) depth distribution of earthquakes associated with T1 and T2 from the EQS-Catalog.

Earthquake data from this paper, integrated with geological data from the literature (Supplementary Fig. S11 and references therein), were used as high-quality constraints to identify and reconstruct three well-distinct non-planar fault models (T1, T1-splay and T2) (Fig. 6).

T1, in the portion corresponding to the ABT trace as drawn in Fig. 2, has an average along-strike length of 210 km and an along-dip length (i.e., width) of ~ 85 km at depths from 1 to 32 km. It is characterized by a mean N240° dip-azimuth and 20° dip-angle (Fig. 6a and Supplementary Fig. S14).

T1-splay detaches from T1 at a depth of about 20 km (Fig. 3d) and reaches the surface along the trace of the Near Coast Thrust (NCT, Fig. 1c) with mean N236° dip-azimuth and 25° dip-angle (Fig. 6a).

T2, corresponding to a hidden thrust, has an average along-strike length of ~ 150 km and an along-dip length (*i.e.*, width) of ~ 80 km at depths from − 25 km to − 60 km. It has a mean N226° dip-azimuth and 24° dip-angle (Fig. 6a,b and Supplementary Fig. S14). On average, T1 and T2 are 12.5 km apart (Fig. 6c).

The T1 and T2 surfaces are given as depth contour lines in Fig. 6d,e. The depth contour lines show that T1 rotates from NW-SW to near N–S along the strike; in particular, the depth contour lines between 20 and 25 km, in the proximity of the Sarnano locality, show a sharp NNE–SSW right-lateral bend (Fig. 6d), which appears kinematically coherent with a cluster of associated strike-slip FMs. The latter belongs to SS-Family1 (Fig. 2b).

Nearly 90% of earthquakes from EQS-Catalog associated with T1 are located at depths between 4 and 22 km; 90% of those associated with T2 are located at depths between ~ 20 and 50 km (Fig. 6f).

## Discussion

Earthquakes at lower crust depths (> ~ 20 km) and even at upper mantle depths are uncommon within the continental lithosphere. However, in collisional settings, we can find seismic events at such depths beneath the India-Tibet collision zone^50^, and more recently, they were highlighted within the European continental crust beneath the northern foreland of the Central Alps^51^ as well as within the Adria continental crust, beneath portions of the outermost Apennine thrust belt from the Padan region to Sicily^13,52,53^ and beneath the Outer Dinarides belt^54^.

The triggering mechanisms are still poorly understood and generically referred to as the presence of a strong lower crust layered with thin mafic to ultramafic, sill-like intrusions, of high-pressure fluids^51,55^.

The results obtained in this paper provide additional constraints on the occurrence of the lower crust and upper mantle seismicity within the continental lithosphere, offering a detailed geometric reconstruction necessary for the realistic modeling of the geodynamic context. Furthermore, as modeled for the Himalayan belt^56^, fault friction and non-planar fault geometry may strongly control the earthquake cycle, the segmentation pattern, and the possible occurrence of bimodal seismicity, with important implications for future 3D SHA calculations.

The EQS- and FMS-Catalogs (2009–2017) allowed us to add essential elements to reconstruct the eastern Central Italy's complex seismotectonic compressional framework, showing that also background seismicity is a valuable tool to delineate faults with confidence. Furthermore, the availability of high-quality microseismicity relocations and focal mechanisms, together with many geologic sections and seismic lines (Supplementary Fig. S11) and the availability of a deep-crust seismic reflection profile (Fig. 3b, and “2D analysis of earthquake/fault association”), make the study area a pivotal zone to constrain the lithospheric-scale geometry, kinematics, and stress-field of the ongoing deformation along the Adriatic outer front of the Apennines in Central Italy.

### T1 and T2 geometry: a novel configuration

Along the coastal Adriatic area (eastern Central Italy), a westward deepening of the seismic activity from the upper crust to ~ 70 km beneath the Apennines is known in the literature and associated with the activity of the outer Apennine thrust front, referred to as ABT^18,29^, but also as active basal thrust decollement^57^ or as shallow slab of the Adriatic lithosphere^6,33,58^.

The 3D geometry of such structure (T1 in the present paper) has been recently reconstructed at depths from 1 to 17 km by Petricca et al.^59^, mainly based on seismic lines, and at depths from 8 to 40 km by DISS Working Group^31^. The high-quality earthquake data presented in this paper constrain a novel and more detailed and complex geometry with events focusing on two distinct principal thrust planes (T1 and T2), on average 12.5 km apart (Fig. 6a).

T1 has an average dip-angle of ~ 20° versus ~ 11° in Petricca et al.^59^ and ~ 5° in DISS Working Group^31^. The T1 earthquake distribution from EQS-Catalog is not homogeneous along-dip. It is concentrated at the upper crust (< ~ 10 km, ~ 10%), lower crust (10–28 km, ~ 85%), and, subordinately, Moho depths (28–35 km, ~ 5%). A similar layering of seismicity is also outlined in the Padan area, where the seismicity is concentrated within the Mesozoic multilayer, at the basement top, and Moho depths^6,13^.

T2 has an average dip-angle of ~ 24°; ~ 65% of the associated events are within the lower crust (20–40 km), and the remaining 30% are at upper lithospheric depths (40–60 km) (Fig. 4). The T1 and T2 earthquake depth distribution are coherent with the thermo-mechanical properties and stratification of the lithosphere^18,48^. In particular, the sub-crustal seismicity, almost exclusively associated with T2, is supported by the high strength values (21 ± 6 TN/m) of the Adriatic mantle lithosphere^48,60^.

The presence at the outer Apennine thrust front of two distinct regional scale seismogenic volumes, e.g., T1 and T2, can open a discussion on alternative geodynamic scenarios whose demonstration is beyond the scope of this paper; nonetheless, we introduce some elements to foster the discussion.

On one side, T1 and T2 could be interpreted as earthquake features typical of Double Seismic Zones (DSZs, sensu^61,62^) associated with a subduction zone, that can present along-strike variation^63^ and eventually merge at depth^64^. Spacing between DSZs planes is variable (usually 15–35 km) and temperature-dependent (colder slab-larger spacing)^65^.

However, most commonly and worldwide, the DSZs occur at intermediate depths (~ 70–350 km), within the lower lithosphere and, mostly, in the mantle asthenosphere^64^; usually, down-dip reverse fault earthquakes prevail in the upper plane and down-dip normal fault ones in the lower plane^65^. In our Italian study case, the two seismic planes are both confined within the lithosphere (depths < 60 km), have a narrow spacing (12–15 km), and both present a coaxial shortening axis.

Conversely, T1 and T2 could be interpreted as two lithospheric thrusts displacing and shortening the Adriatic continental lithosphere. In such a context, T2 would represent the outermost thrust of the foreland-ward propagating Apennine belt with a thick-skinned style^18^. We observe as the map- and section-view distance between T1 and T2 (Figs. 3c and 6) well fit the time–space progression of thrust inception calculated by Basili and Barba^66^ (2–2.5 mm/year) in Central Italy.

In such a context, we speculate whether T2 could represent the down-dip prosecution of the basal thrust of the Mid-Adriatic Ridge (MAR in Fig. 1b), a Late Pliocene–Quaternary fold-and-thrust system located in the central Adriatic Sea and a more external position with respect to the ABT^17^. A critical point of this interpretation is the lack of upper-crust earthquakes associated with T2, illuminating a possible connection between T2 and MAR, and the controversial age of MAR.

The available national earthquake catalogs^28^ show a similar earthquake distribution beneath the Padan regions, implying a possible further extent of the T1-T2 thrust configuration also in Northern Italy (see Supplementary Figs. S1, S2 and S15).

### Multi-depth non-coaxial stress fields

The prevalent deformation regime (reverse or wrench faulting) of the fold-and-thrust belt at the ABT hanging wall is still debated in the literature. Although active shortening perpendicular to the ABT is well supported by morphotectonic^67–69^ and GPS data^25,70,71^, constraints from focal mechanisms^42,43,72^ are more controversial due to the presence of strike-slip mechanisms for some of the major sequences (i.e., Cesena-Forlì 1993 and Ancona 1972).

The presence of these FMs has been variously interpreted in the literature. According to some Authors^73^, the strike-slip deformation is local, subordinate to the regional compressional regime, and due to orocline bending of the arcuate belt. According to others^74^, it is the expression of dominating wrench-faulting regime, with ENE-WSW left-lateral crustal strike-slip faults dissecting the Apennine thrust belt. Still, other Authors^75^ propose an important right-lateral strike-slip deformation in the area south of the Conero promontory.

The analysis of the new focal solution (FMS-Catalog), integrated with the others available in the literature (Figs. 2b, 6b, and Supplementary Fig. S10), clearly shows the need for a 3D approach to address the problem. A crust-scale radial compressional regime associated with both T1 and T2 is certainly the dominant feature shown from FMS data in this paper.

Nevertheless, local clusters with strike-slip kinematics are present. They can be attributed to two spatially and kinematically distinct families, (SS-Family 1 and 2), characterized by different σ_1_-axis orientations. SS-Family 1 is spatially associated with the ABT and its hanging wall splays; SS-Family 2 is much deeper^6^ and located within the T2 footwall volume.

The SS-Family 1 shows SW-NE trending nearly horizontal P-axes approximately coaxial with those of the reverse fault regimes and they are located north of Ancona and at lower crustal depth beneath the Apennine Foothill, close to the Sarnano locality. This latter group corresponds with a local bending of the ABT, as independently revealed by the T1 depth contour lines (Fig. 6d). We observe that SS-Family 1, which is characterized by systems of N–S right-lateral and E–W left-lateral faults syn-kinematic with coeval reverse ones, is a typical subsidiary feature that often develops in a fold-and-thrust belt to accommodate the progressively along-strike bending of the plicative structures during a progressive compressional deformation^18^. This configuration has long been recognized in the outcropping and well-exposed Umbria-Marche fold-and-thrust system^18^, located westward of the studied area.

Conversely, the deep SS-family at the T2 footwall shows NNW–SSE trending P-axes incompatible with those related to the ABT contraction and rather coaxial with the regional wrench tectonics characterizing the Adriatic foreland^6,76,77^ and the footwall of the Apennine thrust sheet, under the mountain chain axial zone^78^. Such a strike-slip deformation has been interpreted as an independent process of the Apennine's progressive eastward migration and shortening and instead associated with Nubia-Adria plate NNW–SSE relative convergence^79,80^. Our data support such an interpretation and extend northward the presence of the deep strike-slip field confined beneath T2 to the latitude of Ancona-Rimini.

When also considering the inner active stress field characterizing the upper crust of the central Apennines^45^, the 3D picture of coexisting (neighboring) deformation volumes undergoing well-distinguished stress fields at different depths becomes rather complex but defined.

We reconstruct two different multi-depth vertical stress configurations, especially well evident across the central sector of the study area (Fig. 6b). Beneath the Apennine belt, we observe from top to bottom the following stress regimes:

1. upper crust tension with SW–NE σ_3_-axis (as in Ferrarini et al.^81^),
2. T1-related lower-crust compression with SW–NE σ_1_-axis,
3. T2-related lower crust-upper mantle (< 60 km) compression coaxial with T1.

Beneath the Coastal area, we observe from top to bottom:

1. T1-related upper crust compression with SW–NE σ_1_-axis,
2. T2-related lower-crust compression with SW–NE σ_1_-axis,
3. deep-SS lower crust wrench tectonics with NW–SE σ_1_-axis.

With the coexistence in a narrow horizontal and vertical space of discrete deformation zones, each with a characterizing stress tensor, the possibility of stress interaction and triggering among them should be considered.

## Conclusions

The new catalogs provided in this paper are helpful for significantly improving the knowledge of 3D geometry, kinematics, and state of stress of the active seismogenic deformation at the outer front of the Apennine thrust belt, in eastern Central Italy.

The overall seismicity distribution clearly shows the seismotectonic complexity of the study area with distinct and overlapping seismogenic volumes: a well-known upper crust extensional one, two compressive ones at depth from the upper crust to the upper mantle, and a subordinate lithospheric strike-slip one. Unlike the extensional domain, capable of releasing earthquakes of magnitude up to ~ 7.0, and the Adriatic Basal Thrust associated with events of maximum magnitude of ~ 6.5, we cannot state or exclude that T2 could release significant earthquakes. However, it might participate in future ruptures with clear implications for seismic hazard evaluation.

Indeed, the two well-distinct lithospheric compressional seismic volumes (T1 and T2), first recognized and reconstructed in detail in this paper, represent a geometric-kinematic fundamental constraint to discuss the Apennine fold-and-thrust system's geodynamic context as a shallow subduction zone^6,82^ or as an intra-continental lithosphere shear zone^83,84^. However, this last geodynamic point is not the target of our paper as it deserves additional investigations and an enlargement of the study area to the overall Padan-Adriatic Arc.

## Methods

### Seismic relocation and focal mechanism solutions

The relocation was performed using the probabilistic nonlinear global search inversion approach (NonLinLoc^32^), considering the 3D Vp and Vp/Vs propagation model optimized for the study area^33^ (Supplementary Text S1). Special attention was paid to inserting reliable station corrections obtained as mean residuals by the location of a set of stable and redundant phase events. The events showing the higher number of phases were chosen on a 3D regular grid (size 5 km) to avoid a non-uniform sampling. The latter (2400 events, 80,358 P phases, 77,135 S phases) were introduced in an iterative procedure, in which the mean residuals of the previous cycle were used as station correction in the next one. After 3 iterations, residuals were stabilized and used for the final locations.

The beachballs were computed using the FPFIT procedure^40^ and adopting the 3D velocity model in Carannante et al.^33^ (Fig. 2b). Quality analysis was performed by using three quality factors (Q), decreasing from A to C, derived from the parameters given by FPFIT code: Qf (degree of polarity misfit), Qp (range of uncertainties of the strike, dip, and rake), and Qstdr (station distribution ratio) (Fig. 2b′).

### Cross sections and earthquake/fault association

The depth distribution of the data from the EQS- and FMS-Catalog (2009–2017), integrated with focal mechanisms from the literature (1967–2009), was analyzed in 2D view along the trace of (1) 6 regional transects with a half-width (i.e., the distance around the transect from which data are included in each section) of 20 km, (2) 23 closely spaced N055°-striking cross-sections, with a half-width of 5 km (Supplementary Fig. S12), (3) 70 radial cross-sections with a half-width 2.5 km (Fig. 3a,c). The radial cross-sections were organized in three sets with directions N040°, N060°, and N080°, to consider the arc shape of the ABT-related compressive structures and ensure an orthogonal projection of the earthquakes and FMs.

Moreover, we projected on the transects the section-view intersections with the traces of the ABT front, its major splay (NCT), the Altotiberina fault (ATF), and the easternmost west-dipping Outcropping Extensional Fault (Fig. 3c). We derived the traces of such geological structures from the Structural Model of Italy (scale 1:500,000), detailed maps and papers, and an extensive compilation of geological sections from the literature (Supplementary Fig. S12 and references therein).

### Strain and stress analysis

Starting from the 180 FMs (the new ones and those collected from the literature), we reconstructed the strain and stress pattern for the eastern Central-Italy crustal volumes and surrounding areas (Fig. 5a–c) to characterize the kinematics of T1, T2, and the strike-slip solutions at T2 footwall (deep-SS).

We performed the spatial analysis of the maximum horizontal stress orientation (SH_max_) associated with T1 and T2 and deep-SS. It represents a single parameter that allows having a simplified stress map and corresponds to the azimuth of P-axes for reverse, reverse-oblique, and a strike-slip solution having a P-, B- and T-axis-plunges less-equal 20°, greater -equal 45° and less than 40° respectively, and the azimuth of T-axes 90° clockwise rotated for the other strike-slip focal mechanisms^41^.

This analysis allowed us to identify kinematically homogeneous sectors characterized by near coaxial SH_max,_ and compute, for each sector, the average focal mechanism representative of T1, T2, and deep-SS.

We built a regular grid (0.1° × 0.1°) and calculated SH_max_ at each node, separately analyzing the FMs associated with T1, T2, and deep-SS. For interpolating the SH_max_, we followed the Carafa and Barba's^48^ approach, which considers the uneven sampling data and the correlation of stress orientation with distance. We used a search radius of 58 km. Since the FMs in some areas are not uniformly distributed along T1 and T2 and deep-SS, we also plotted the reliability of the interpolation considering a maximum permissible uncertainty of 30° (90% of confidence bounds). Based on SH_max_ orientation computed for T1 and T2, we identified three sectors (1 = northern sector, 2 = central sector, and 3 = southern sector; Fig. 5a) in which the stress can be considered homogeneously oriented, and, for each sector, we computed the average FMs representative of the kinematics of T1, T2, and deep-SS. Average FMs were computed using the Bingham statistics (AFM) and the moment tensor summation by weighting the data with the magnitudes (AWFM^85^; Table 1).

To compute the local and regional stress tensors, we independently inverted the FMs falling within sectors 1, 2, and 3 and belonging to the deep SS-Family, obtaining four different stress tensors (Table 2).

We followed the inversion procedure proposed in Delvaux and Sperner^49^, which consists in inverting focal mechanisms for the four model stress parameters σ1, σ2, σ3, and the stress ratio R = (σ2–σ3)/(σ1–σ3). The inversion is optimized by a composite objective function (called F5 in Win-Tensor) of two terms: one depending on the directional part of the resolved shear stress and the other on the magnitudes of resolved normal and shear stress. During the inversion, the procedure simultaneously minimizes the angular misfit between observed and modeled slip on the focal planes (first term) and optimizes the second term by maximizing shear stress magnitudes and minimizing normal stress. In addition, the focal mechanisms are weighted with an exponentially weighted factor (10^M) that depends on the magnitude to give more relevance to the kinematics of larger events.

### 3D fault model building

We adopted a three-step procedure^86–88^ to reconstruct the T1, T1-splay, and T2 non-planar fault models in a 3D georeferenced frame using the Move suite software (Petroleum Experts Ltd., 2020.1).

We first built the T1 and T1-splay shallow-depth surfaces (SdS) by extruding their near-surface traces, schematized as in the map of Fig. 3a, to a depth of 3 km below sea level; the dip-angle was assumed variable along-strike (N040° to N020°) and was derived from an ad hoc GIS compilation of geological sections from literature (Supplementary Fig. S11 with references). Second, in section view, along the transects and the radial sections given in Fig. 3a, we drew curved lines interpolating the hypocentral distributions associated with T1, T1-splay, and T2. Third, we built the 3D non-planar surfaces (Fig. 6a), applying the Delaunay triangulation (Fig. 6c) to interpolate the near fault traces, whenever available, and the section view lines. Finally, the depth contour lines were automatically derived, assuming an initial along-dip spacing of 1 km (Fig. 6d,e).

## Supplementary Information

Supplementary Information 1.

Supplementary Information 2.

Supplementary Information 3.

Supplementary Information 4.

Supplementary Information 5.

Supplementary Information 6.

Supplementary Information 7.

## Data Availability

The original contributions presented in the study are included in the article and the Supplementary Material (Supplementary information 1). All the fields of the data presented in the supplementary material files, (Supplementary information 2=EQS-Catalog.txt; Supplementary information 4=EQS-Catalog_HQ.txt; Supplementary information 5= EQS-Catalog_LQ.txt; Supplementary information 6= FMS-Catalog.txt) are described in the corresponding header files (Supplementary information 3=EQS-Catalog_Header.txt; Supplementary information 7= FMS-Catalog_Header.txt).
