# Supplementary material for: Lithospheric double shear zone unveiled by microseismicity in a region of slow deformation
Source: Sci Rep. 2022 Dec 6;12:21066. doi: 10.1038/s41598-022-24903-1 (PMC9727122; doi:10.1038/s41598-022-24903-1)
Supplement: Supplementary file 1 — Supplementary Information 1. [file 41598_2022_24903_MOESM1_ESM.docx]

**Lithospheric double shear zone unveiled by microseismicity in a region of slow deformation**

Rita de Nardis^1-2*^, Claudia Pandolfi^1-2^, Marco Cattaneo^3^, Giancarlo Monachesi^3^, Daniele Cirillo^1-2^, Federica Ferrarini^1-2^, Simone Bello^1-2^, Francesco Brozzetti^1-2^ & Giusy Lavecchia^1-2^

^1^ DiSPuTer, Department of Psychological, Health and Territorial Sciences, University “G. d’Annunzio” Chieti-Pescara, Chieti, Italy

^2^ CRUST - Centro inteRUniversitario per l’analisi Sismotettonica Tridimensionale, Italy

^3^ INGV Istituto Nazionale di Geofisica e Vulcanologia, Ancona, Italy

*Corresponding author: rita.denardis@unich.it

**Supplementary Material**

**Contents of this file:**

1. Introduction
2. Text S1
3. Figures S1 to S15
4. References

**Additional Supporting Information (Files uploaded separately in ASCII format)**

***Earthquakes occurred in eastern Central Italy from 2009-2017***

EQS-Catalog.txt: Catalog of relocated hypocenter data

EQS-Catalog_HQ.txt: Subset-1 high-quality seismic locations

EQS-Catalog_LQ.txt: Subset-2 lower quality seismic locations

EQS-Catalog_Header.txt: header information of EQS-Catalog

***Focal mechanisms from 2009-2017 within the Marche-Adriatic Contractional Province with earthquake/fault association***

FMS-catalog.txt: Table parameters of fault plane solutions

FMS-Catalog_Header.txt: header information of FMS-Catalog

**1. Introduction**

This supplementary material aims to give further information about the relocation results (EQS-catalog) and the new focal mechanism solutions (FMs-catalog) used in this work and furnish additional figures to support the reliability of T1 and T2 fault models built within the regional seismotectonic context in eastern Central Italy.

Text S1 describes the methodology used to relocate the seismic data and provides a detailed description of the dataset quality (subset-1 and subset-2) we used to build the geometry of T1 and T2.

Figures S1 and S2 show the epicentral and hypocentral distribution in Central Italy as defined by the Italian Seismic Bulletin (ISIDe Working group, 2007). This is functional to give an idea of the quality and resolution of earthquake distribution by comparing those available from national catalogs and our data (EQS-catalog). In these Figures, we divided the seismicity into three-time intervals to show the influence of the National Seismic Network (RSN) on the identification of potentially seismogenic structures. The first interval (Time1) spans from 1985 to 2004; the second one (Time 2) from 2005 to 2009. In 2005, there was an important upgrade of the RSN system (Amato and Mele, 2008), and in 2009, some of the stations of the regional seismic network ReSIICO (2013) were integrated with the National seismic network systems, improving the seismic location in our study area (*e.g.*, eastern Central Italy). For this reason, and to give an element of comparison with EQS-Catalog, the third time interval (2009-2017, Time3) was chosen of the same time length of the EQS-catalog we provide.

Figure S3 schematically shows the main geological structures, the main toponyms present in the text and the main events occurred in the study area both in historical and instrumental time.

Figure S4 shows the geometry of regional seismic network ReSIICO (Monachesi et al., 2013) and the Italian seismic network RSN, which is fundamental for producing the high-quality seismic locations and focal mechanisms of the EQS- and FMs-Catalogs.

Figures S5 and S6 give detailed information on the statistical distribution of the location parameters of data in the EQS-Catalog.

Figures S7 and S8 show the epicentral and hypocentral distribution of the EQS-catalog data subdivided into two sub-sets with different classes of quality parameters.

Figures S9 and S10 show the criteria for the spatial selections of the focal mechanisms in FMS-catalog and compare our solutions with others available in the literature for a subset of events.

Figure S11 shows hypocentral cross-sections from EQS-Catalogue.

Figure S12 is a map view of a set of cross-sections used to build the 3D-models and a compilation of geological and geophysical traces from the literature across the study area.

Figures S13, S14 give additional information on the T1 and T2 fault models' geometry and the associated earthquakes as extracted from EQS-catalog.

Figure S15 opens the question, for further investigation, on the extent of T1 and T2 geological structures along the northern-central Apennines of Italy. It shows the map and section views of seismicity occurring in the northern Apennines, where it is possible to observe an earthquake distribution like the one shown in Figures S1 and S2.

**2. Text S1.** ***Detection, Picking and earthquake locations***

***2.1 Detection and picking***

The data source of the relocated dataset is the ReSIICO seismic network (Monachesi et al., 2013), located in eastern Central Italy (Figure S4). The derived EQS-catalog represents the development of the mixed automatic-manual seismic catalog for eastern Central Italy whose procedure and homogeneity of results are described in Cattaneo et al. (2017).

From 2009 to 2013, the seismic events, recognized by an STA/LTA algorithm, were manually treated by human operators using interactive programs. From 2013 to 2016, the first analysis, after the detection, was performed by the automatic RSNI-Picker described in Spallarossa et al. (2014) and Scafidi et al. (2016); just the events for which the automatic procedure was not able to produce a good-quality location were subjected to a manual revision. In 2017 a new automatic detection and picking procedure was introduced based on the Complete Automatic Seismic Processor (CASP) software (Scafidi et al., 2019): the detection is always based on an STA/LTA analysis, but it is refined by using the Akaike Information Criterion (AIC) function (Akaike, 1974).

***2.2 Picking refinement***

In the latter approach, the station detections are introduced in an event detection module based on coincidence criteria on sub-networks of stations (Spallarossa et al., 2021). The so obtained events are then analyzed by the picking and locating engine RSNI-Picker2 (Scafidi et al., 2018), an evolution of the earlier RSNI-Picker; the main improvements are related to introducing different iteration steps, in which pickings are refined based on computed locations. NonLinLoc software (Lomax et al., 2000), based on a nonlinear global search method, is used for locating events. In case of non-convergence of the automatic procedure, a manual picking intervention was required.

***2.3 Station correction***

The whole dataset of picked phases was subjected to a relocation procedure to produce a homogeneous catalog. The core of this procedure is the NonLinLoc software; this program allows to adopt both 1-D and 3-D velocity models and apply station corrections. We first inverted the P and S phases using the 1D velocity model proposed in De Luca et al (2009), and then a 3D model as in Carannante et al. (2013). It is worth noting that the 1D model is the starting model used by Carannante for the 3D inversion. The mean residuals obtained by the location of a set of stable and redundant phase data were considered for both the 1D and the 3D models, as a proxy for the best station corrections.

To this purpose, from the whole dataset, a subset was extracted, choosing for each mesh of a 3D regular grid, the events showing the higher number of phases. This subset (2400 events, 80358 P phases, 77135 S phases) was introduced in an iterative procedure, in which the mean residuals of the previous cycle were used as station correction in the next cycle. After three iterations, residuals were stabilized; mean residuals coming from stations showing a high enough number of samples were used in the final locations. This procedure shows that station corrections were relatively small along the Apennine chain, mainly for the P phases. The largest corrections were needed for stations installed in the Adriatic foreland, both for P and S waves; this result is coherent with the tomographic inversion results in Carannante et al. (2013), showing very low-velocity values for the shallowest layers in this area.

***2.4 Final catalog***

The final phases dataset consisted of 4370652 P phases and 4052053 S phases. These phases were introduced in the location procedure, using, at first, the adopted 1D (De Luca et al., 2009), then the 3D (Carannante et al., 2013) model jointly to the computed station corrections. As a result, we obtained 239298 seismic locations from which we eliminated the non-tectonic events, as defined in Cattaneo et al. (2014). Indeed, in this area quarry blasts and other non-tectonic signals can contaminate our catalogs, mainly in areas where the detection threshold of the seismic network allows to locate low-magnitude events. The catalog of non-tectonic events, defined in Cattaneo et al. (2014) for the period 1996-2012, has been continuously updated by the team managing the ReSIICO network so that it was possible to subtract from our catalogs the events classified as non-tectonic by this analysis.

The final locations were then submitted to a quality check; following the suggestions of Husen and Smith (2004), in addition to the usually adopted statistical error estimates of the location instability (usually referred to as *Error H* and *Error Z,* error estimates for the horizontal and vertical coordinates)*,* NonLinLoc produces traditional Gaussian estimates such as the expectation hypocenter location. The distance between the maximum likelihood and expectation hypocenter locations can represent a good estimate of the stability of the location itself. We use 4 geometrical parameters to define the stability of our locations. In fact, in addition to *Error H* and *Error Z,* we also considered *distH* and *distZ* (defined as the horizontal distance between maximum likelihood and expectation hypocenter locations and the relevant vertical distance, respectively) (Figure S6). For the following analysis, we discard all the locations exceeding 3 km for *Error H* and *distH*, 5 km for *Error Z* and *distZ*.

Among the selected events, we further distinguish the more stable part as the events showing *Error H, Error Z, distH,* and *distZ* all below 1 km. We defined two subsets (Figures S5 and S6): subset-1 containing high-quality relocations and subset-2 a low-quality one. The number of events of subset-1 is reduced to 140637 (see EQS-Catalog_HQ), while subset-2 is composed of 27940 (EQS-Catalog_LQ) earthquakes. As possible to observe from Figures S5 and S6, although we have defined subset-2 as lower quality, it is still rather stable being *Error H* and *distH* between 1 and 3, *Error Z* and *distZ* between 1 km and 5 km.

**3. Figures with captions**

***
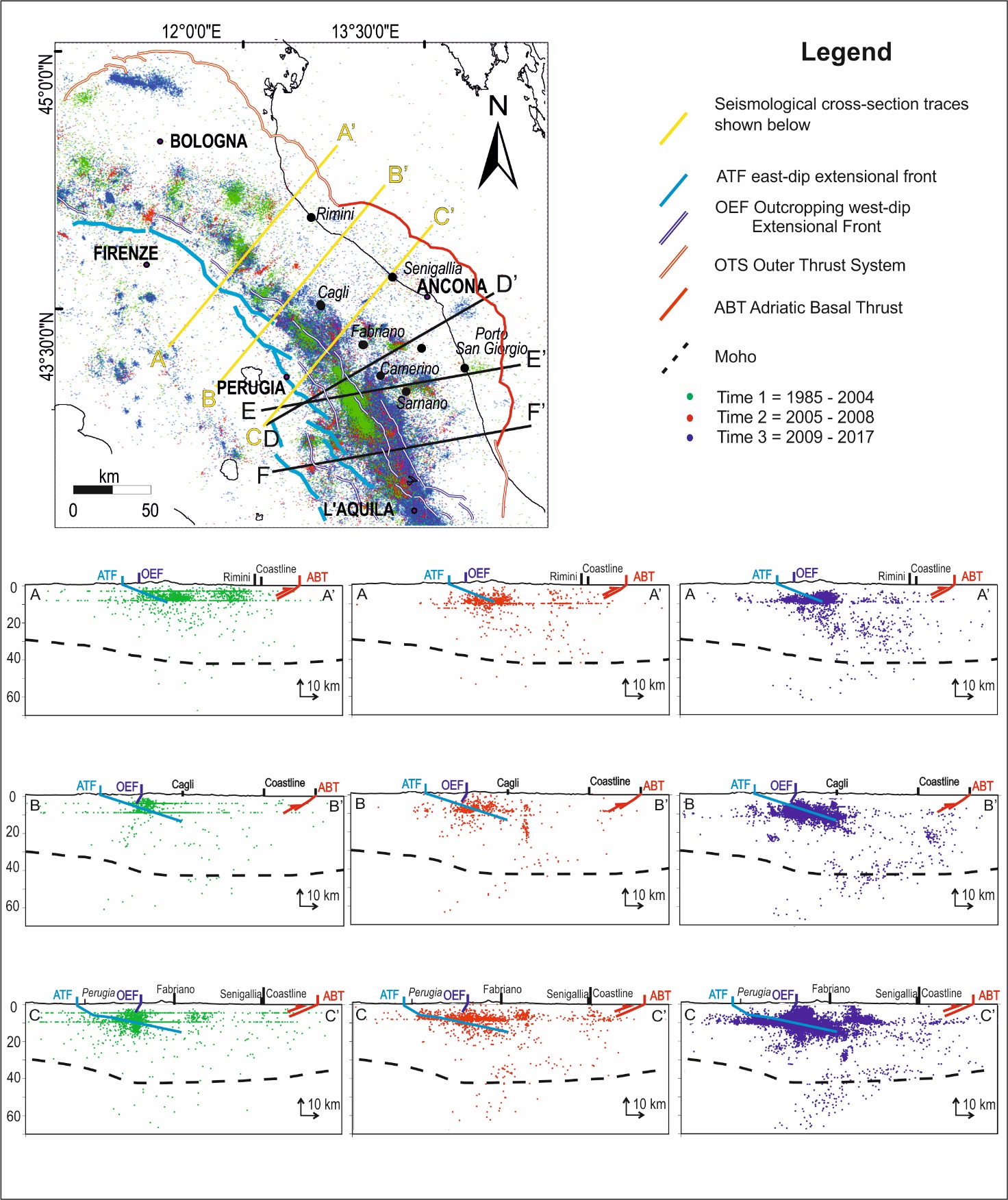
***

**Figure S1.** Map and section view (AA’, BB’, CC’) of the instrumental seismicity from the Italian Seismological Instrumental and parametric Database (ISIDe Working group, 2007) plotted in different time intervals (T1=1985-2004; T2=2005-2008; T3=2009-2017). Each section view shows earthquakes projected within 20 km of half-width. The Moho depth line is from Di Stefano et al. (2011).

**
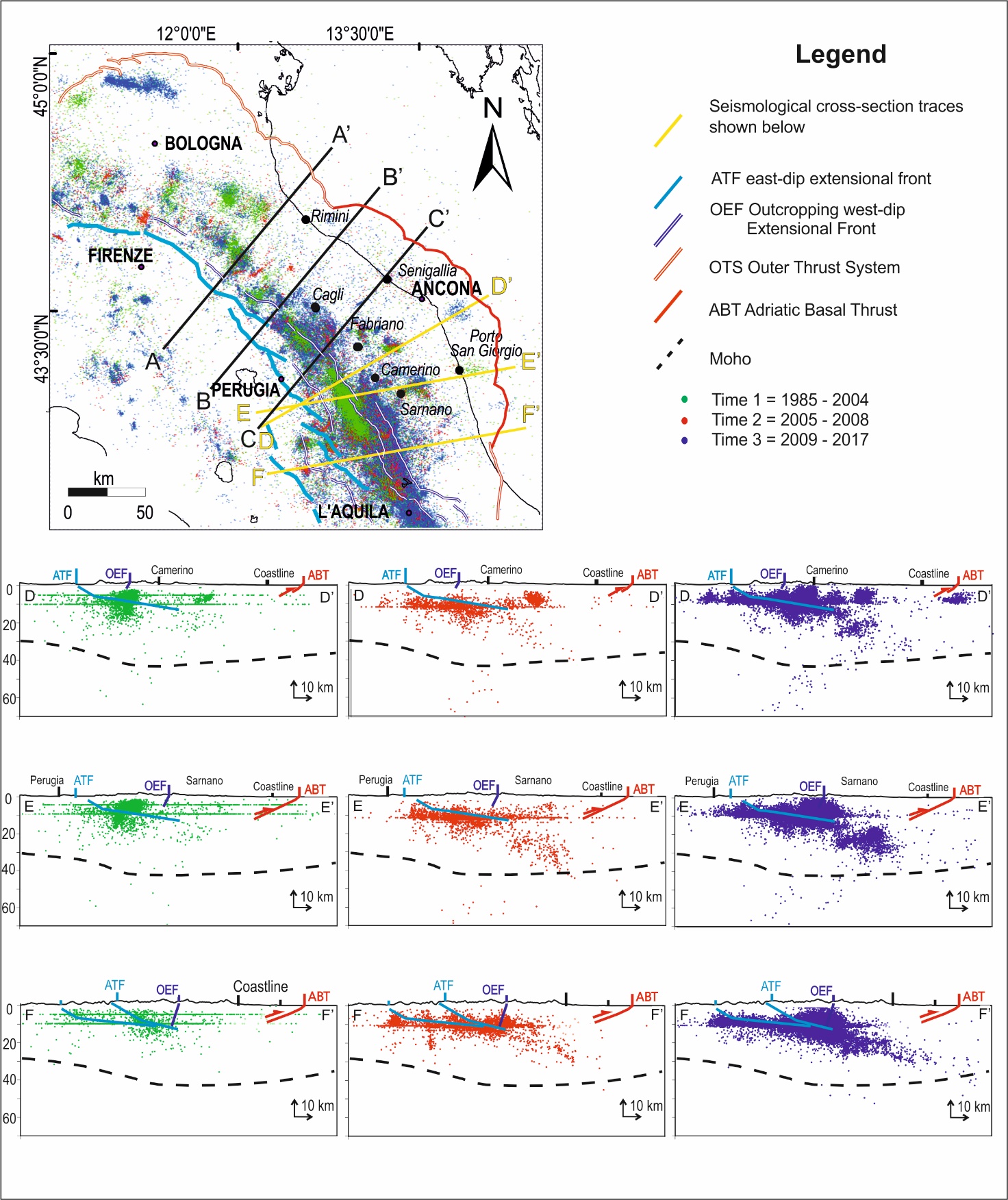
**

**Figure S2.** Map view and section view (DD’, EE’, FF’) of the instrumental seismicity from the Italian Seismological Instrumental and parametric Database (ISIDe Working group, 2007) plotted in different time intervals (T1=1985-2004; T2=2005-2008; T3=2009-2018). Each section view shows earthquakes projected within 20 km of half-width. The Moho depth line is from Di Stefano et al. (2011).

**
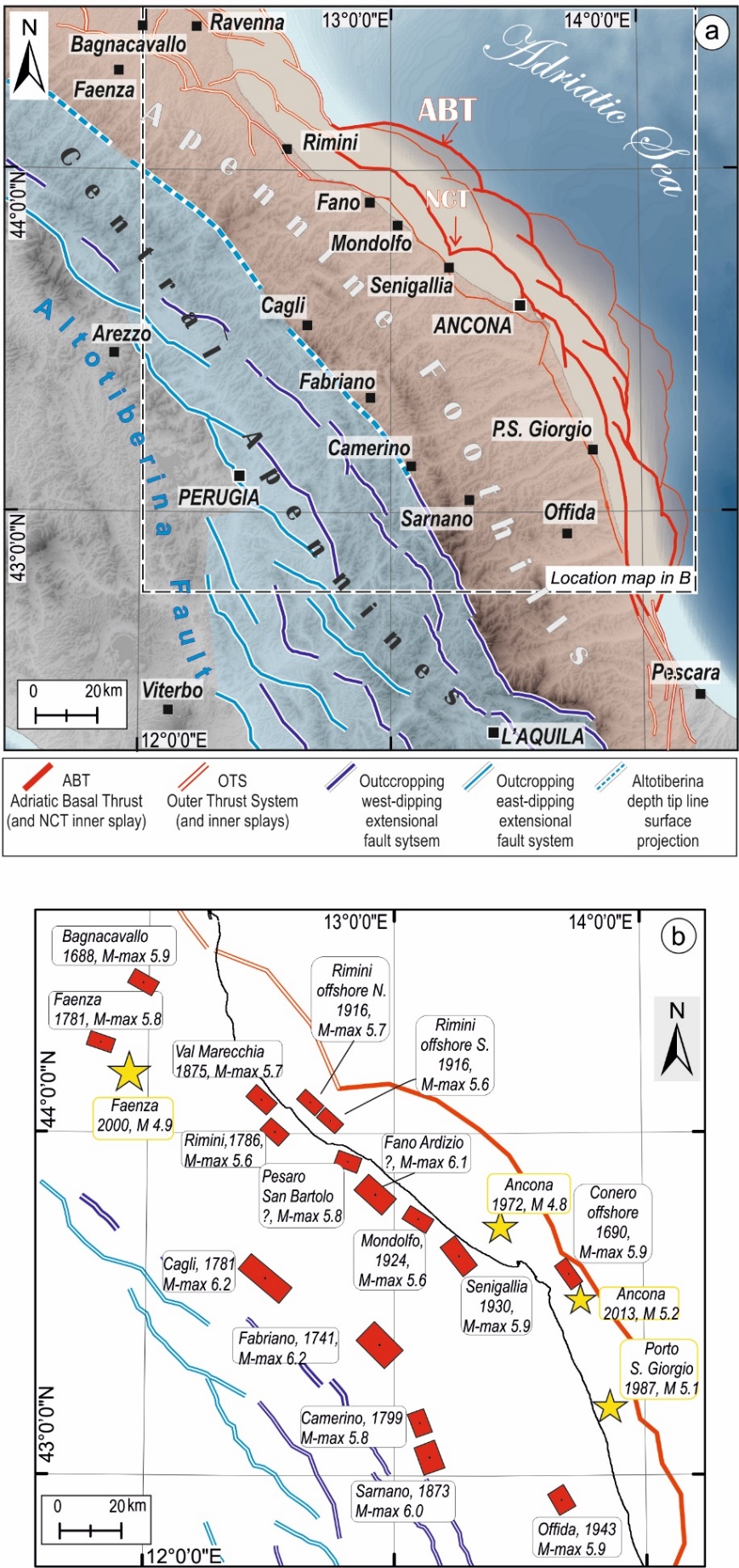
**

**Figure S3.** a) The Adriatic Basal Thrust and surrounding structures with toponyms cited in the main text. NCT (Near Coast Trust) is the major ABT splay. b) Map showing the historical and instrumental seismicity of the study area. Seismogenic boxes (red rectangular symbols) as in the Database of Individual Seismogenic Sources (DISS, Working Group 2021), and location (yellow stars) of main instrumental seismic sequences. The name of the box, the date of the latest earthquake associated with it and the maximum magnitude given to the seismogenic source are labeled white near the box. The labels of instrumental earthquakes, embedded in the yellow rectangles, are referred to the toponym, year, and the magnitude of the main event of the seismic sequence as in (Rovida et al., 2020, 2021).


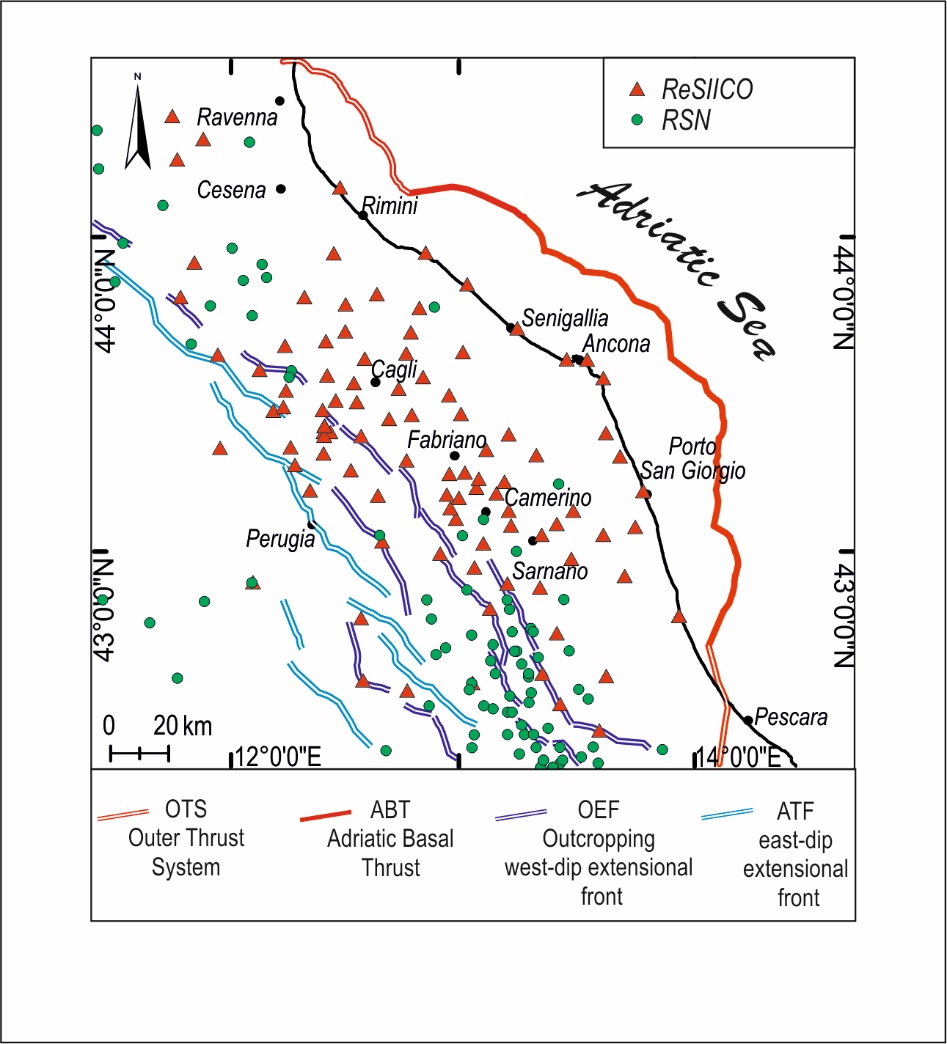


**Figure S4.** Map of seismic stations used for the relocation of seismicity occurred in eastern Central Italy from 2009 to 2017. The red triangles represent the stations of the ReSIICO (Rete Sismometrica dell’Italia Centro Orientale) regional permanent network (Monachesi et al., 2013) and the green circle symbols the ones of the Italian Seismic Network (RSN), respectively. This integrated network consists of 101 seismic stations, 49 of which are equipped with velocimeters, 24 with accelerometers and 28 with both (Marzorati et al., 2016). Phases (Cattaneo et al., 2019a) and seismic locations (Cattaneo et al., 2019b) recorded by ReSIICO can be found at the following link: http://www.an.ingv.it/ReSIICO/index.php

***
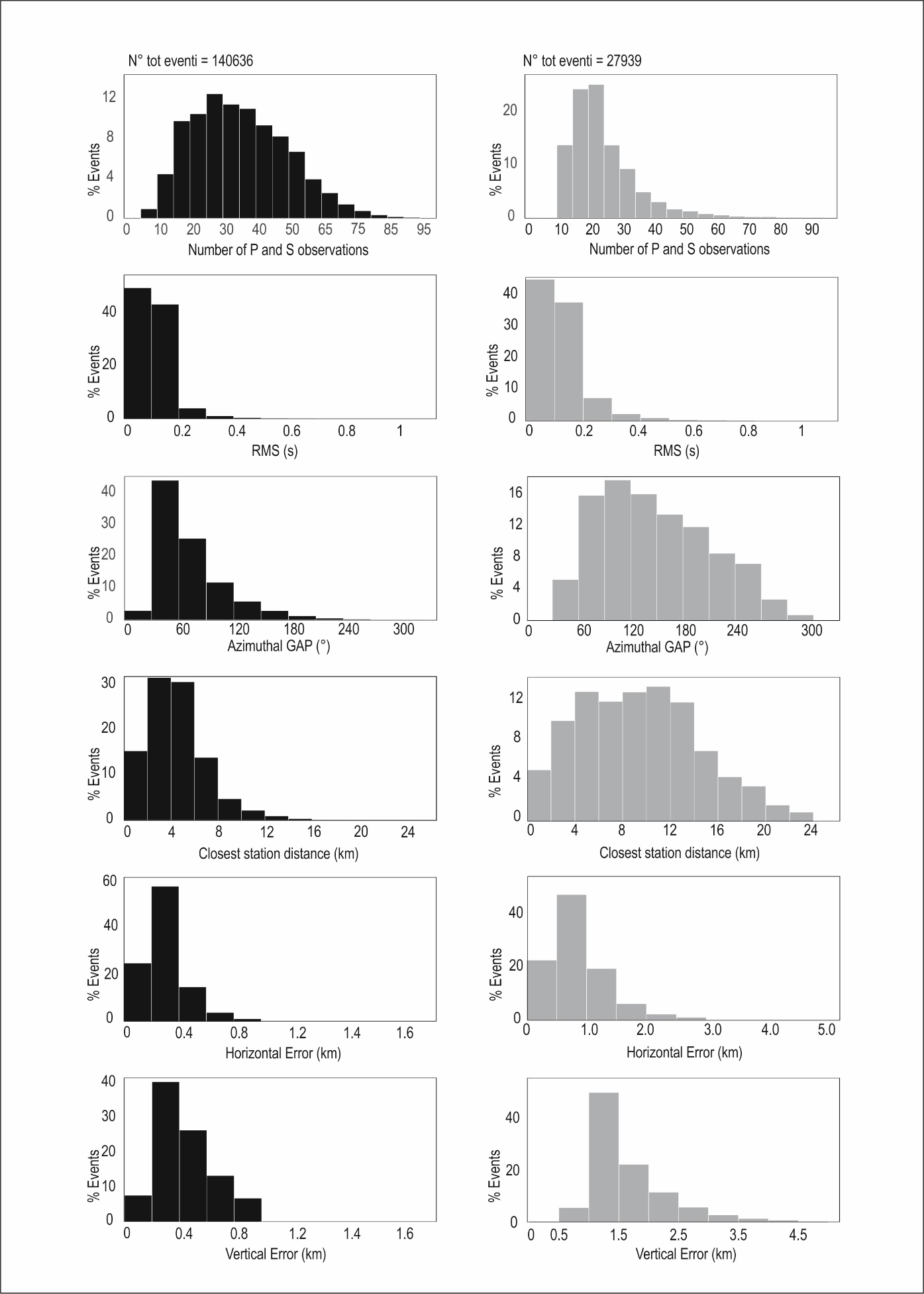
***

**Figure S5.** Statistical distribution of the location parameters of subset-1 (black bars) and subset-2 (grey bars).

**
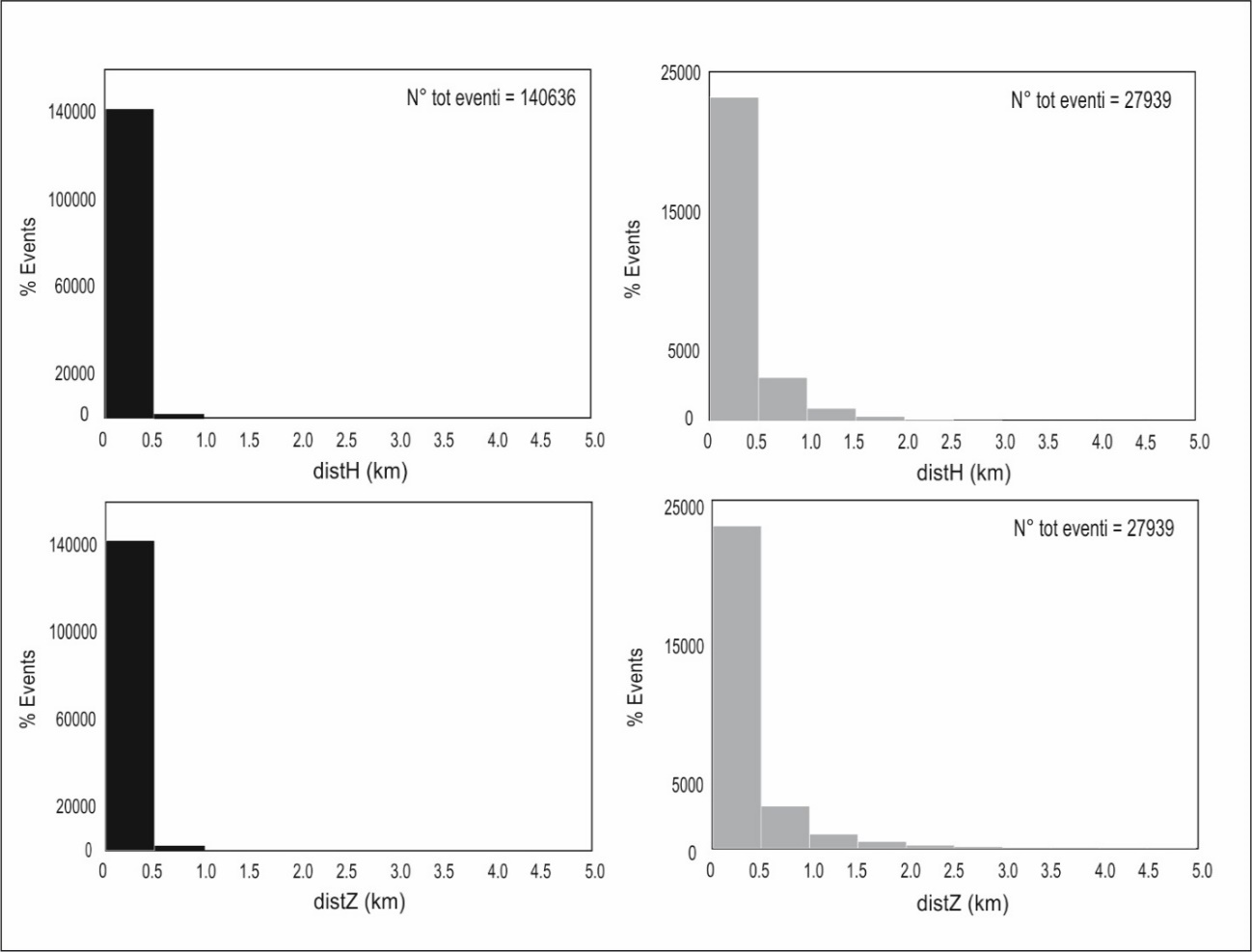
**

**Figure S6.** Statistical distribution of the location parameters of subset-1 (black bars) and subset-2 (grey bars). Key: distH= horizontal distance between the maximum likelihood and expectation hypocentre locations; distZ= Vertical distance between the maximum likelihood and expectation hypocentre

***
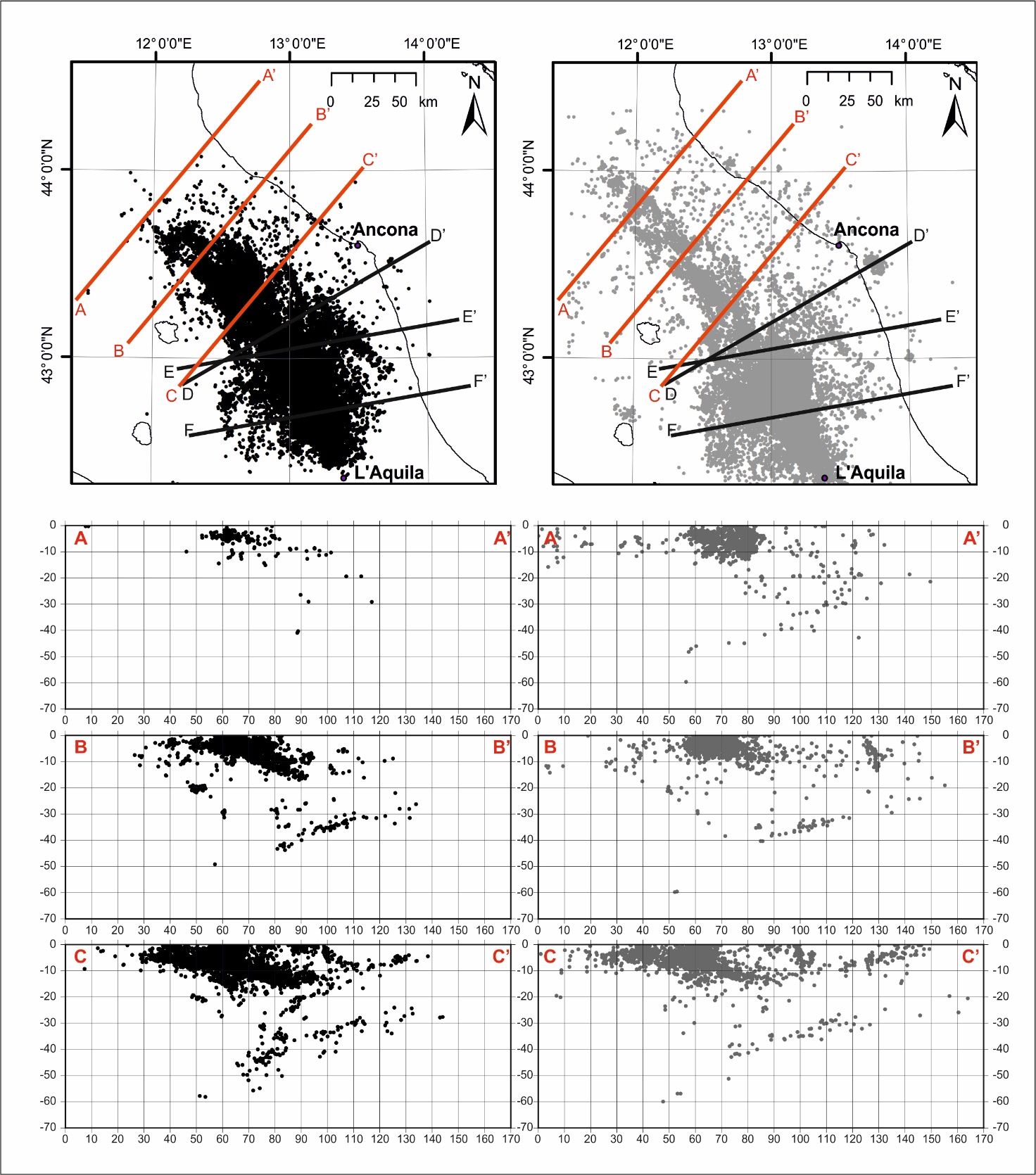
***

**Figure S7.** Maps views and cross-sections (AA’, BB’, CC’) of the events belonging to subset-1 (black dots) and subset-2 (grey dots) having two different classes of quality parameters (see Text S1 and Figure S5-S6 for further details).

***
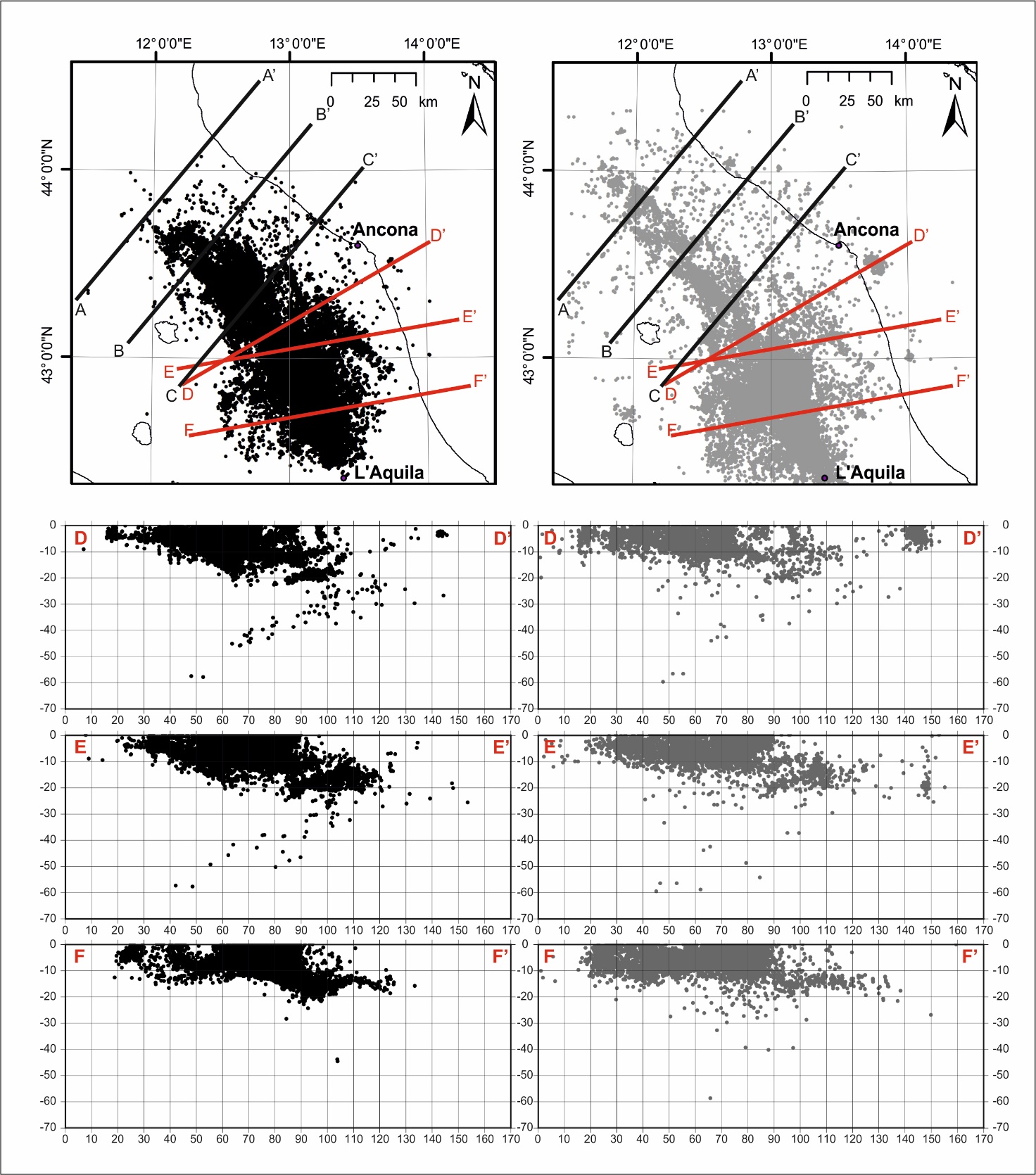
***

**Figure S8.** Maps views and cross-sections (DD’, EE’, FF’) of the seismic events belonging to subset-1 (black dots) and subset-2 (grey dots) having two different classes of quality parameters (see Text S1 and Figure S5-S6 for further details).


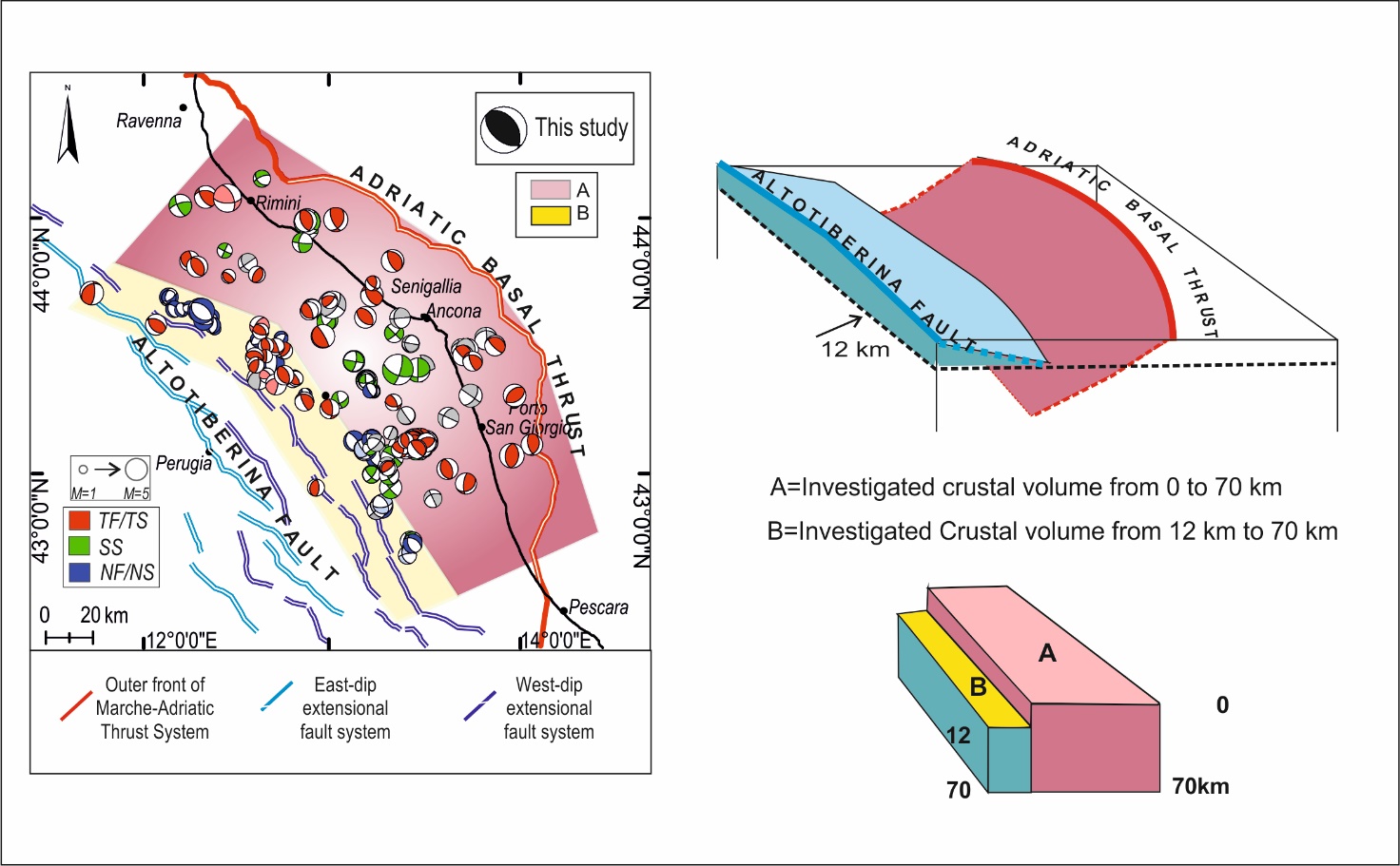


**Figure S9.** Sketch showing the areal extent and volumetric boundaries of crustal volumes investigated to select the focal mechanisms.


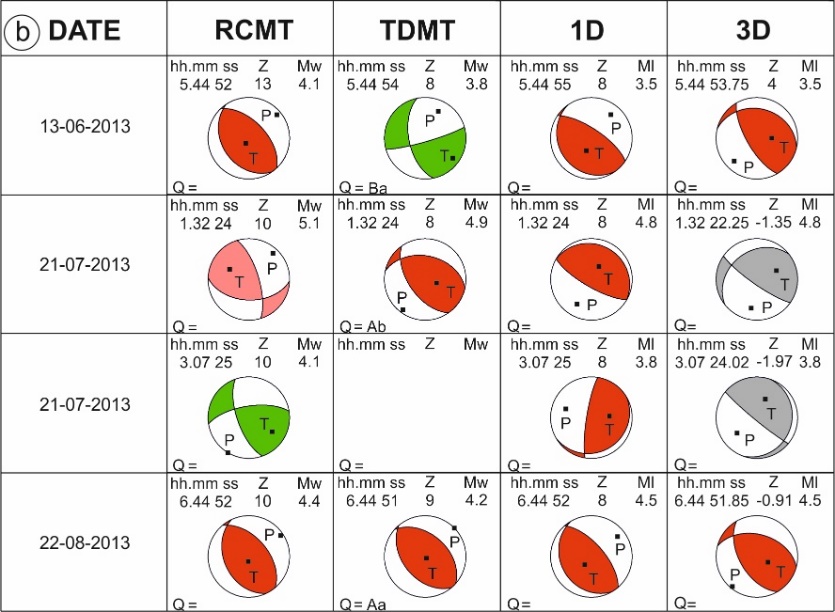

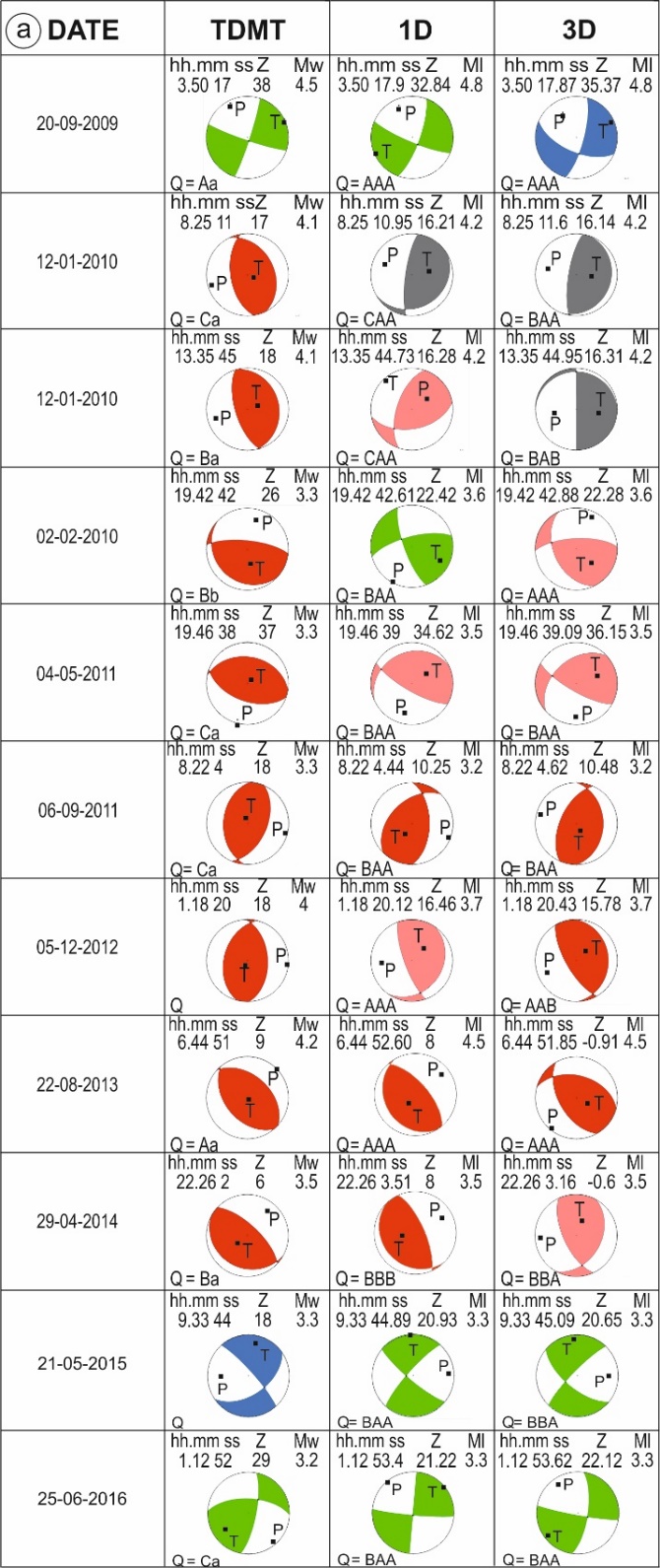


**Figure S10.** (a) Comparison among focal mechanism solutions available from TDMT database (Time Domain Moment Tensor, Scognamiglio et al., 2006), the ones obtained by first motion polarities and 1D velocity model (1D, Monachesi et al., 2012) and this study (3D). (b) Comparison among focal mechanism solutions of the major events related to 2013 seismic sequence. Key: RCMT= Regional Centroid-Moment Tensors (Pondrelli et al., 2006); TDMT= Time Domain Moment Tensor (Scognamiglio et al., 2006); 1D= FMS obtained by first motion polarities and 1D velocity model (Monachesi et al., 2012), 3D= This Study.


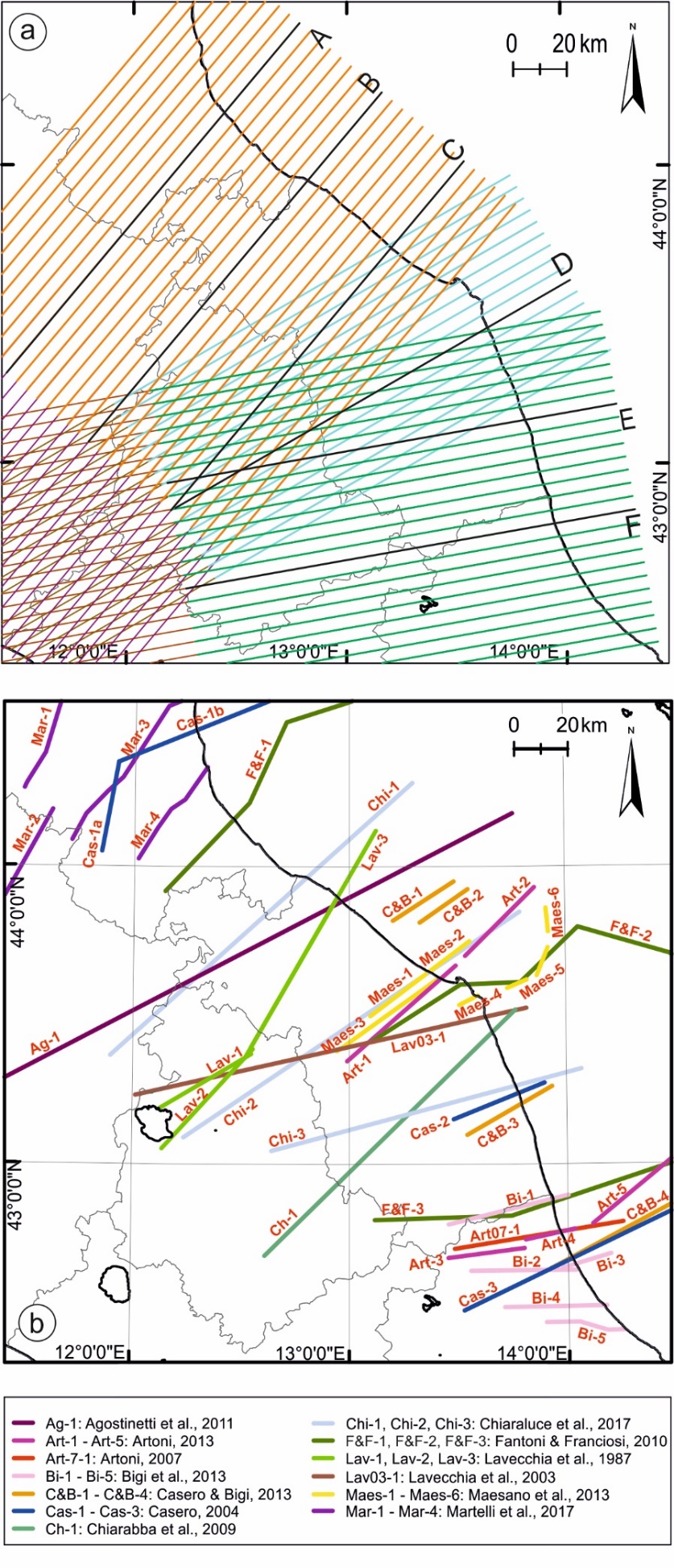


**Figure S11.** Location map of seismological, geological, and geophysical cross-sections used in this study. a) 70 radial cross-sections used to build the 3D model; b) Geological and geophysical cross-sections available in the literature which were the primary source of information used to reconstruct the major fault alignments.


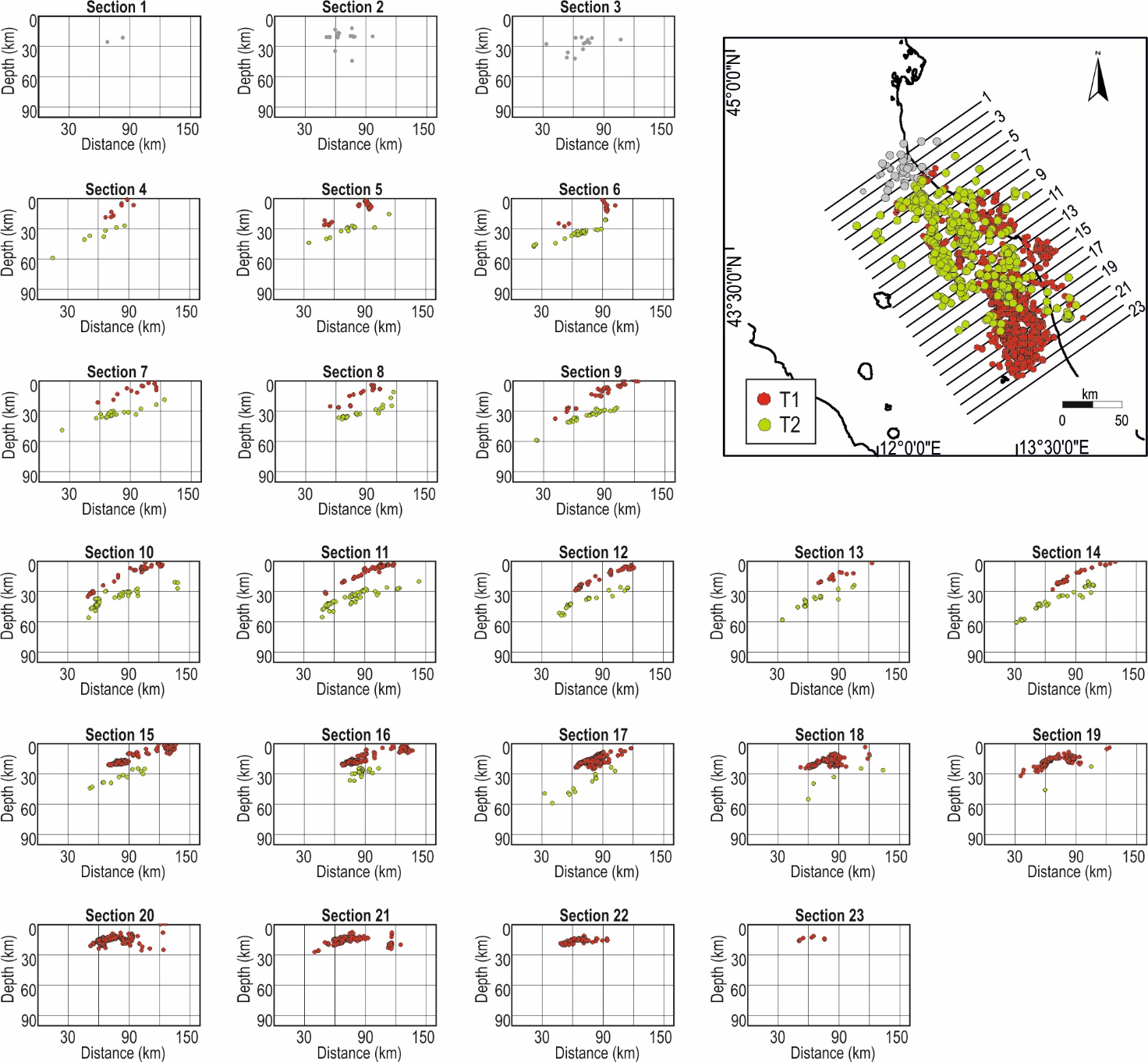


**Figure S12.** Examples of serial cross-sections used for 2D and 3D identification and definition of T1 and T2 geometry. Red and green circles represent the earthquakes (from EQS-Catalog) associated to T1 T2, respectively. The grey circles are the events with undefined association.


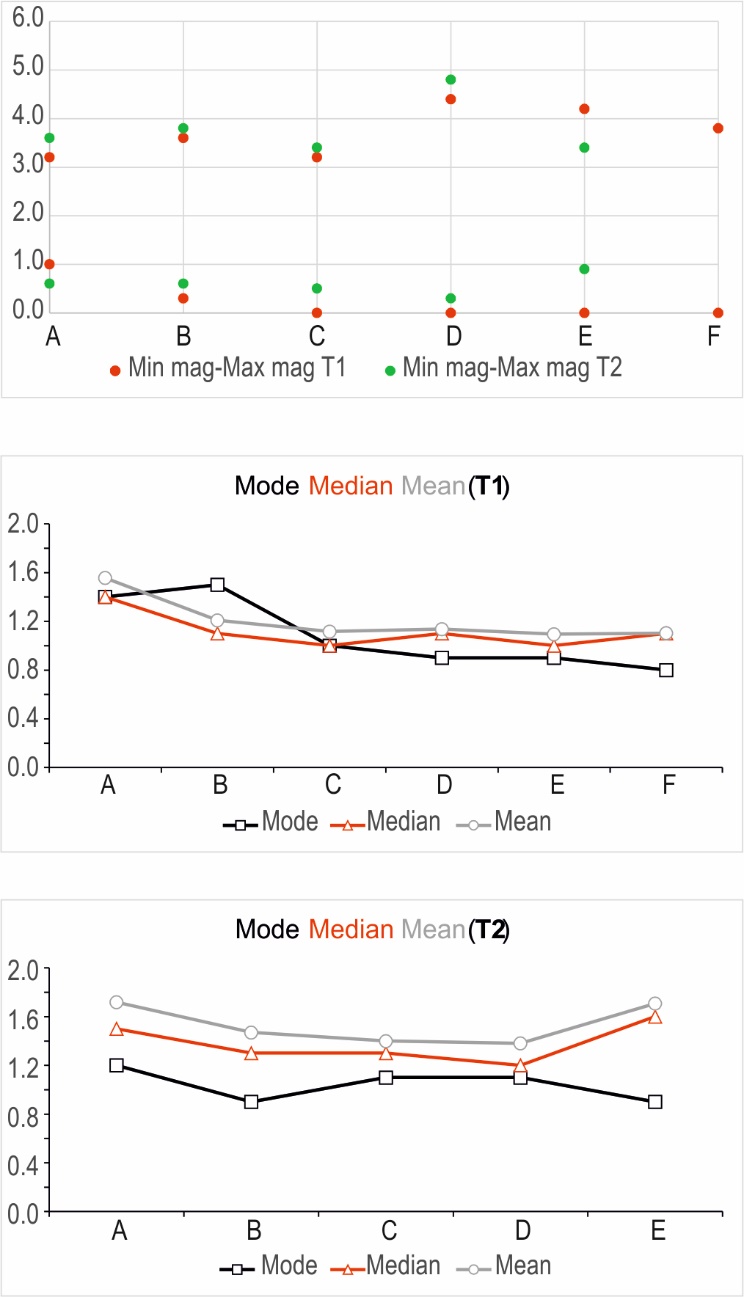


**Figure S13.** Characterization of the magnitude of seismicity along each cross section for T1 and T2. Upper panel represents the minimum and maximum magnitude, the last two panels represent the median, mode and mean of the magnitude distribution of events falling within a half-width of 20 km.


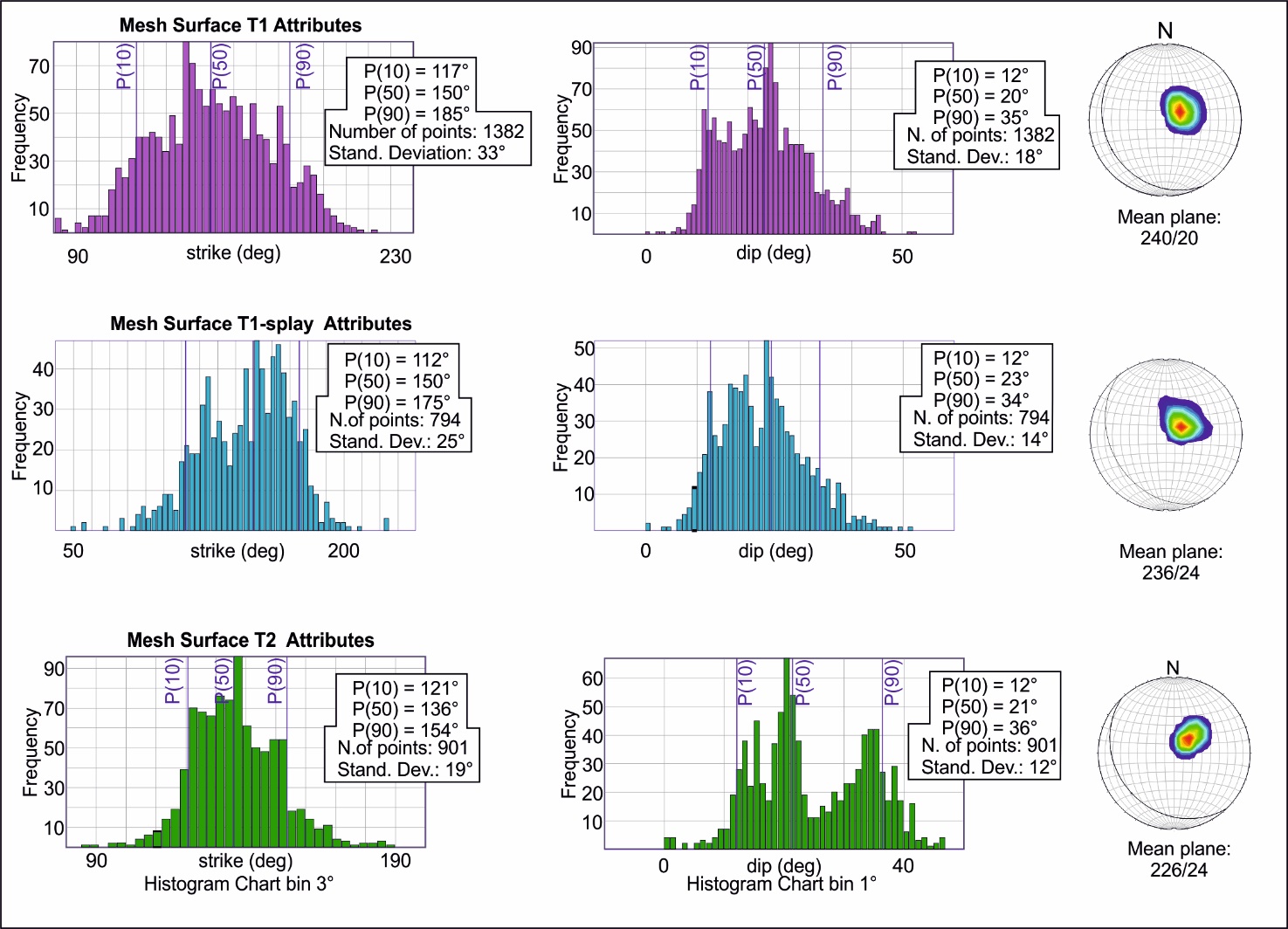


**Figure S14**. Geometric analysis of fault surfaces T1, T1-splay, and T2. The attitude of the fault meshes of the grid sampling of each surface is analyzed with the MOVE Petrex software (Petroleum Experts, 2021) and represented as strike and dip histograms and as stereonet plots of the pole to the meshes attitudes (Lambert azimuthal equal-area projection, lower hemisphere). Percentile values (PE) at 10%, 50%, and 90% are reported together with the mean values and their standard deviation.

**
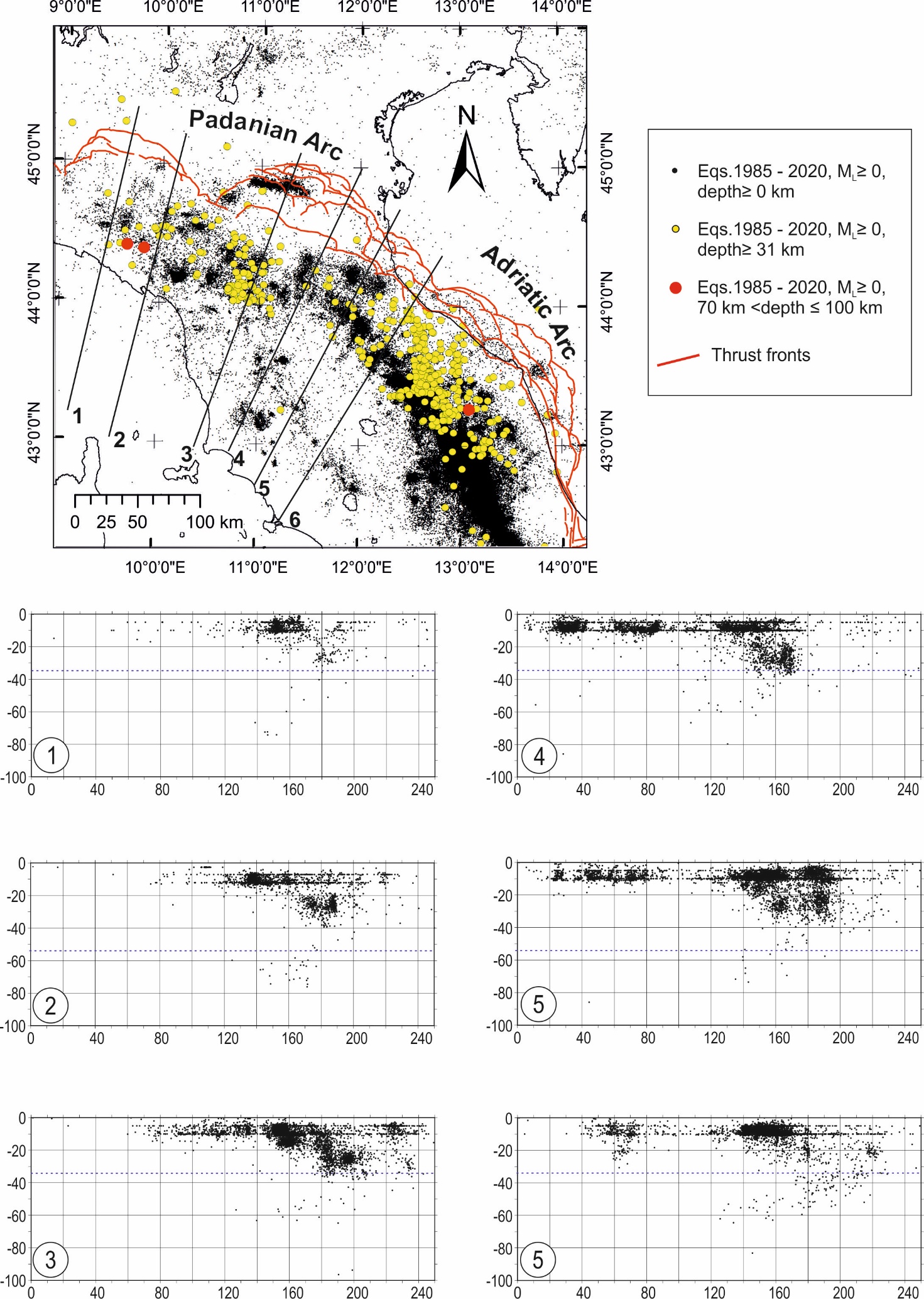
Figure S15.** Map and cross-sections of seismicity derived from Italian Seismic Bulletin data (ISIDe Working group, 2007) along the northern Apennines of Italy.

**4. References**

Agostinetti, N. P., Bianchi, I., Amato, A., Chiarabba, C., 2011. Fluid migration in continental subduction: The Northern Apennines case study. Earth Planet. Sci. Lett., 302, 267–278.

Akaike, H., 1974. Markovian representation of stochastic processes and its application to the analysis of the autoregressive moving average process. Ann. Inst. Stat. Math., 26, 363–387.

Amato, A., Mele, F., 2008. Performance of the INGV National Seismic Network from 1997 to 2007, Ann. Geophys. 51, 417-431.

Artoni, A., 2007. Growth rates and two-mode accretion in the outer orogenic wedge-foreland basin system of Central Apennine (Italy). Boll. Soc. Geol. It. (Ital. J. Geosci.), Vol. 126, No. 3, pp. 531-556.

Artoni, A., 2013. The Pliocene-Pleistocene stratigraphic and tectonic evolution of the Central sector of the Western Periadriatic Basin of Italy. Mar. Pet. Geol., 42, 82-106.

Bigi, S., Conti, A., Casero, P., Ruggiero, L., Recanati, R., Lipparini, L., 2013. Geological model of the central Periadriatic basin (Apennines, Italy). Mar. Petrol. Geol., 42, 107-121.

Carannante, S., Monachesi, G., Cattaneo, M., Amato, A., Chiarabba, C., 2013. Deep structure and tectonics of the northern-central Apennines as seen by regional-scale tomography and 3-D located earthquakes. J. Geophys. Res., 118, 5391–5403.

Casero, P., 2004. Structural setting of petroleum exploration plays in Italy, in: Crescenti, V., D.’Offizi, S., Merlino, S., Sacchi, L. (Eds.), Geology of Italy, Special volume of the Italian Geological Society for the IGC 32 Florence-2004, pp. 189-199.

Casero, P., Bigi, S., 2013. Structural setting of the Adriatic basin and the main related petroleum exploration plays. Mar. Petrol. Geol., 42, 135-147.

Cattaneo, M., Caffagni, E., Carannante, S., D’Alema, E., Frapiccini, M., Ladina, C., Marzorati, S., Monachesi, G., 2014. A catalogue of non-tectonic earthquakes in central-eastern Italy. Ann. Geophys., 57/3, DOI: 10.4401/ag-6434.

Cattaneo M., Monachesi G., Frapiccini M., Calamita C., Pantaleo D., Carluccio I., Ladina C., Marzorati S. 2019a. ReSIICOphs. Database of the Central Eastern Italy Seismometric Network: phases [Data set]. Istituto Nazionale di Geofisica e Vulcanologia (INGV). <https://doi.org/10.13127/resiico/phs>

Cattaneo M., Monachesi G., Frapiccini M., Calamita C., Pantaleo D., Carluccio I., Ladina C., Marzorati S., 2019b. ReSIICOeqs. Database of the Central Eastern Italy Seismometric Network: locations [Data set]. Istituto Nazionale di Geofisica e Vulcanologia (INGV). <https://doi.org/10.13127/resiico/eqs>

Cattaneo, M., Frapiccini, M., Ladina, C., Marzorati, S., Monachesi, G., 2017. A mixed automatic-manual seismic catalog for Central-Eastern Italy: analysis of homogeneity. Annals of Geophysics 60, S0667.

Chiarabba, C., De Gori, P., Speranza, F., 2009. Deep geometry and rheology of an orogenic wedge developing above a continental subduction zone: Seismological evidence from the northern-central Apennines (Italy). Lithosphere, 1(2), 95–104.

Chiaraluce, L., Barchi, M. R., Carannante, S., Collettini, C., Mirabella, F., Pauselli, C., Valoroso, L., 2017. The role of rheology, crustal structures and lithology in the seismicity distribution of the northern Apennines. Tectonophysics, 694, 280–291.

De Luca, G., Cattaneo, M., Monachesi, G., Amato, A., 2009. Seismicity in the Umbria-Marche region from the integration of national and regional seismic networks. Tectonophysics, 476, 219–231.

Delvaux, D., Moeys, R., Stapel, G., Petit, C., Levi, K., Miroshnichenko, A., 1997. Paleostress reconstructions and geodynamics of the Baikal region, Central Asia. Part II: Cenozoic rifting. Tectonophysics, 282(1-4), 1–38. <https://doi.org/10.1016/S0040-1951(97)00210-2>

DISS Working Group, 2021. Database of Individual Seismogenic Sources (DISS), Version 3.3.0: A Compilation of Potential Sources for Earthquakes Larger Than M 5.5 in Italy and Surrounding Areas. Available at: <http://diss.rm.ingv.it/diss/>, Istituto Nazionale di Geofisica e Vulcanologia.

Fantoni, R., Franciosi, R., 2010. Tectono-sedimentary setting of the Po Plain and Adriatic foreland. Rend. Fis. Acc. Lincei, 21(1), 197–209. <https://doi.org/10.1007/s12210-010-0102-4>.

Husen, S., Smith, R., 2004. Probabilistic earthquake location in three dimensional velocity models for the Yellowstone National Park region, Wyoming, Bull. seism. Soc. Am., 94(6), 880–896

ISIDe Working Group, 2007. Italian Seismological Instrumental and Parametric Database (ISIDe). Istituto Nazionale di Geofisica e Vulcanologia (INGV). doi:10.13127/ISIDE Scognamiglio et al., 2009

Lavecchia, G., Minelli, G., Pialli, G., 1987. Contractional and extensional tectonics along the transect Lake Trasimeno-Pesaro (Central Italy), in: Boriani et al. (Eds), The Lithosphere in Italy Advances in Earth Science Research. Italian Mid-Term Conference on the Lithosphere Program, Atti Conv. Lincei, 80, 177-194.

Lavecchia, G., Boncio, P., Creati, N., 2003. A lithospheric-scale seismogenic thrust in Central Italy. J. Geodyn., 36, 79-94.

Lomax, A., Virieux, J., Volant, P., Berge-Thierry, C., 2000. Probabilistic earthquake location in 3D and layered models: introduction of a Metropolis–Gibbs method and comparison with linear locations, in Advances in Seismic Event Location, eds Thurber, C.H. & Rabinowitz, N., pp. 101–134. Kluwer Academic Publishers.

Maesano, F. E., Toscani, G., Burrato, P., Mirabella, F., D’Ambrogi, C., Basili, R., 2013. Deriving thrust fault slip rates from geological modeling: Examples from the Marche coastal and offshore contraction belt, Northern Apennines, Italy. Mar. Petrol. Geol., 42, 122-134.

Martelli, L., Bonini, M., Calabrese, L., Corti, G., Ercolessi, G., Molinari, F. C., Piccardi, L., Pondrelli, S., Sani, F., Severi, P., 2017. Carta sismotettonica della Regione Emilia-Romagna e aree limitrofe. Con Note illustrative. Regione Emilia-Romagna, Servizio geologico, sismico e dei suoli. D.R.E.AM. Italia.

Marzorati, S., Cattaneo, M., Frapiccini, M., Monachesi, G., Ladina, C., 2016. Recent seismicity before the 24 August 2016 Mw 6.0 central Italy earthquake as recorded by the ReSIICO seismic network. Annals of Geophysics 59, 1-12.

Mildon, Z. K., Roberts, G. P., Faure Walker, J. P., Iezzi, F., 2017. Coulomb Stress Transfer and Fault Interaction over Millennia on Non-planar Active normal Faults: the Mw6.5-5.0 Seismic Sequence of 2016-2017, central Italy. Geophys. J. Int. 210 (2), 1206–1218. doi:10.1093/gji/ggx213

Monachesi, G., Cattaneo, M., Ladina, C., Marzorati, S., Frapiccini, M., Carannante, S., Ferretti, M., Sebastianelli, M., Delladio, A., Selvaggi, G., 2013. Esperienze di monitoraggio integrato: il caso della Rete Sismometrica dell’Italia Centro Orientale, di quella Accelerometrica Marchigiana e dei suoi servizi. Quaderni di Geofisica, 106, 1-30 ISSN:1590-2595

Monachesi, G., Marzorati, S., Ladina, C., Cattaneo, M., Frapiccini, M., D'Alema, E., Carannante, S., 2012. Beach Balls in central western Italy. The focal mechanisms of the Earthquakes recorded by the RESIICO (central oriental Italy seimological network). Istituto Nazionale di Geofisica e Vulcanologia. http://www.an.ingv.it/BB/home.html Accessed 14 August 2021

Pondrelli, S., Salimbeni, S., Ekström, G., Morelli, A., 2006. The Italian CMT Dataset From 1977 to the Present. Phys. Earth Planet 159, 286–303. doi:10.1016/j.pepi.2006.07.008

Rovida A., Locati M., Camassi R., Lolli B., Gasperini P., 2020. The Italian earthquake catalogue CPTI15. Bulletin of Earthquake Engineering, 18, 2953-2984.

Rovida, A., Locati, M., Camassi, R., Lolli, B., Gasperini, P., Antonucci, A., 2021. Italian Parametric Earthquake Catalogue (CPTI15), Version 4.0 (Italy: Istituto Nazionale di Geofisica e Vulcanologia (INGV)). doi:10.13127/CPTI/CPTI15.3

Petricca, P., Carminati, E., Doglioni, C., 2019. The Decollement depth of active thrust faults in italy: implications on potential earthquake magnitude. Tectonics, 38. <https://doi.org/10.1029/> 2019TC005641.

Scafidi, D., Spallarossa, D., Ferretti, G., Barani, S., Castello, B., Margheriti, L., 2019. A complete automatic procedure to compile reliable seismic catalogs and travel-time and strong-motion parameters datasets. Seismol. Res. Lett. 90(3), 1308–1317.

Scafidi, D., Spallarossa, D., Turino, C., Ferretti, G., Viganò, A., 2016. Automatic P and S-Wave Local Earthquake Tomography: Testing Performance of the Automatic Phase-Picker Engine “RSNI-Picker”. Bull. Seism. Soc. Am., doi: 10.1785/0120150084

Scafidi, D., Viganò, A., Ferretti, G., Spallarossa, D., 2018. Robust picking and accurate location with RSNI-Picker2: real-time automatic monitoring of earthquakes and non-tectonic events, Seismol. Res. Lett, 89, 1478– 1487.

Scognamiglio, L., Tinti, E., Quintiliani, M., 2006. Time Domain Moment Tensor [Data Set]. Istituto Nazionale di Geofisica e Vulcanologia (INGV). doi:10.13127/TDMTsdata.2018.49

Spallarossa, D., Cattaneo, M., Scafidi, D., Michele, M., Chiaraluce, L., Segou, M., Main, IG., 2021. An automatically generated high-resolution earthquake catalogue for the 2016–2017 Central Italy seismic sequence, including P and S phase arrival times. Geophysical Journal International, 225, 555–571, <https://doi.org/10.1093/gji/ggaa604>.

Spallarossa, D., Ferretti, G., Scafidi, D., Turino, C., Pasta, M., 2014. Performance of the RSNI-Picker. Seism. Res. Lett., 85/6, doi:10.1785/0220130136.
